# Dissecting clinical and biological heterogeneity in clinical states of bipolar disorder: a 10-year retrospective study from China

**DOI:** 10.3389/fpsyt.2023.1128862

**Published:** 2023-12-21

**Authors:** Ting Zhu, Ran Kou, Yao Hu, Minlan Yuan, Cui Yuan, Li Luo, Wei Zhang

**Affiliations:** ^1^West China Biomedical Big Data Center, West China Hospital, Sichuan University, Chengdu, China; ^2^Med-X Center for Informatics, Sichuan University, Chengdu, China; ^3^Business School, Sichuan University, Chengdu, China; ^4^Mental Health Center of West China Hospital, Sichuan University, Chengdu, China; ^5^Sichuan Provincial Center for Mental Health, The Center of Psychosomatic Medicine of Sichuan Provincial People’s Hospital, University of Electronic Science and Technology of China, Chengdu, China

**Keywords:** bipolar disorder, biological heterogeneity, clinical states, machine learning, electronic medical records (EMR), Shapley additive explanations (SHAP)

## Abstract

**Objectives:**

To dissect clinical and biological heterogeneity in clinical states of bipolar disorder (BD), and investigate if neuropsychological symptomatology, comorbidity, vital signs, and blood laboratory indicators are predictors of distinct BD states.

**Methods:**

A retrospective BD cohort was established with data extracted from a Chinese hospital’s electronic medical records (EMR) between 2009 and 2018. Subjects were inpatients with a main discharge diagnosis of BD and were assessed for clinical state at hospitalization. We categorized all subjects into manic state, depressive state, and mixed state. Four machine learning classifiers were utilized to classify the subjects. A Shapley additive explanations (SHAP) algorithm was applied to the classifiers to aid in quantifying and visualizing the contributions of each feature that drive patient-specific classifications.

**Results:**

A sample of 3,085 records was included (38.54% as manic, 56.69% as depressive, and 4.77% as mixed state). Mixed state showed more severe suicidal ideation and psychomotor abnormalities, while depressive state showed more common anxiety, sleep, and somatic-related symptoms and more comorbid conditions. Higher levels of body temperature, pulse, and systolic and diastolic blood pressures were present during manic episodes. Xgboost achieved the best AUC of 88.54% in manic/depressive states classification; Logistic regression and Random forest achieved the best AUCs of 75.5 and 75% in manic/mixed states and depressive/mixed states classifications, respectively. Myocardial enzymes and the non-enzymatic antioxidant uric acid and bilirubin contributed significantly to distinguish BD clinical states.

**Conclusion:**

The observed novel biological associations with BD clinical states confirm that biological heterogeneity contributes to clinical heterogeneity of BD.

## Highlights

- Mixed states showed more severe suicidal ideation and psychomotor abnormalities, while depressive states showed more common anxiety, sleep, and somatic-related symptoms.
- Higher levels of body temperature, pulse, and systolic and diastolic blood pressures were present during BD manic episodes.
- Myocardial enzymes and the non-enzymatic antioxidant uric acid and bilirubin contributed significantly to distinguish BD clinical states.

## 1 Introduction

Bipolar disorder (BD) refers to a severe mood disorder affecting more than 1% of the global population and is associated with a high socio-economic burden (1). BD is characterized by alternating episodes of mania/hypomania (elevation of mood and increased energy and activity) and depression (lowering of mood and decreased energy and activity) (1). A subset of BD patients may currently exhibit either a mixture or a rapid alteration of manic and depressive symptoms, which is defined as mixed state (2). Mixed states require individuals to meet both the diagnostic criteria for depression and mania. The Diagnostic and Statistical Manual of Mental Disorders, 5th edition (DSM-5) has supplanted mixed states with the mixed features specifier, which requires the presence of at least 3 depressive (manic or hypomanic) non-overlapping symptoms during a hypomanic or manic (major depressive) episode (3). Akiskal et al. (4) argue that depressive symptomatology also is common among those diagnosed with BD manic episode; however, the symptoms are insufficient to meet the criteria for BD mixed episodes. BD is a complex disease with heterogeneous clinical manifestations, and traditional categorical paradigm of BD spectrum (manic, depressive, and mixed states) typically has different disease courses, treatment responses, and prognoses (5).

The diagnosis of BD is made by a comprehensive clinical assessment, however, there is no biomarker (such as genetic testing) that can inform the diagnosis, prognosis, or treatment outcome of BD (3, 6). In clinical practice, major challenges persist recognizing BD and mixed state is prone to misdiagnosis as other clinical states, due to widely varying rates of individual symptoms and overlapping symptoms (7). The clinical implications of detecting mixed states are that antidepressants should not be prescribed to adults with mixed states of BD (8–10). Difficulties in the accurate differential diagnosis impede the effective treatment of patients, which may worsen the prognosis (11) and increase the risk of switching between different states (12, 13). There is also substantial evidence of the negative effects of cumulative episodes on cognitive function, somatic and psychiatric comorbidity (14), and high suicide rate (15). Therefore, in order to improve the early diagnosis of BD, there is an urgent need to better dissect clinical and biological heterogeneity in different BD clinical states. While much attention has been given to differential diagnosis of BD and other psychiatric disorders [such as major depressive disorder (MDD), attention-deficit hyperactivity disorder (ADHD), borderline personality disorder, substance use disorders, and schizophrenia (SCZ)] (3), few works have started to investigate whether shared biological risk explaining some of the overlapping psychopathological symptoms in different BD clinical states exist (16). Part of the reason is the lack of objective diagnostic markers and targeted criteria to comprehensively assess different BD clinical states, and systematically obtained data on biological characteristics and the spectrum and severity of symptomatology are limited (17).

Previous analyses of BD clinical states focused on comparing two samples (manic and mixed states) across demographic differences, symptom presentations, treatment patterns (18), and personality features (16). Studies on sleep and circadian heart rate rhythms showed to be sensitive to BD clinical states (19). Bipolar mood states (euthymic state, depressive state, and mixed state) can also be related to electrodermal tonic activity (20). Singh et al. (21) assessed the spectrum and severity of bipolar symptoms that differentiated BD mixed state from BD-depression or BD-mania/hypomania, employing the Bipolar Inventory of Symptoms Scale (BISS). In addition, laboratory non-enzymatic antioxidants (including uric acid, bilirubin, and albumin) were reported that they can be used as markers to reflect the level of oxidative stress, and their serum concentrations may be associated with the onset of bipolar disorder (22). A recent meta-analysis reported that uric acid levels were higher in BD manic episodes vs. BD depressive episodes (23). Some studies have found that a panel of urine metabolites (24, 25) and other blood biomarkers (26) could prove a promising path for the search of biomarkers in BD, but biomarkers useful in distinguishing different BD clinical states have not yet been established. Although the above studies provide important insights into several aspects of BD, whether it is mixed, manic, or depressive, there is little focus on comparing the differences in clinical, physiological, and biological characteristics of these three diagnoses simultaneously. It remains unclear whether the corresponding link between clinical states and overlapping heterogeneity could be demonstrated by neuropsychological symptomatology, vital signs, comorbidity, and blood laboratory indicators. To our knowledge, no study has comprehensively compared the clinical status of BD and attempted to develop objective evaluation measures to establish a data-driven diagnostic decision support model. The purpose of this study is to help fill this gap.

Current studies in BD have been hampered by small sample sizes, but electronic medical records (EMR) provide an exciting opportunity for large-scale clinical studies at low cost and with high classification accuracy (27). With the increasing availability of EMR, high fidelity heterogeneous data on patient information has been captured during hospitalization care, yet the utilization of this data to improve patient diagnosis and quality of care remains poor. Particularly, unstructured data such as narrative notes in EMR often record substantial clinical details about patients’ current condition and symptoms, which may be easily accessible and have not previously been used in machine learning (ML) models. Most commonly, clinicians organize information gathering around their patients’ self-report of current or most recent mood state, and then present it in the chief complaint. Therefore, natural language processing (NLP) technology could be a promising method to parse narrative notes in EMR data, focusing on identifying and extracting patients’ symptoms and current mood states. Recent works have shown that ML models are well suited to differentiate BD from MDD using MRI data and blood biomarker data (28–30). No application of interpretable ML methods and NLP techniques to analyze EMR data for the identification and classification of different clinical states of BD has been found. To address this unmet need, we leverage recent advances in medical informatics and present new ML methods to aid in dissecting clinical and biological heterogeneity in clinical states of BD and in providing interpretable key markers in ML models that differentiate patient-specific states.

The aims of the present study were (1) ascertain whether BD patients showed different patterns of vital signs, comorbidities, bipolar symptoms, and blood laboratory indicators, depending on their clinical state, (2) determine which markers might be helpful to differentiate BD clinical states, (3) develop ML models to accurately distinguish BD clinical states before treatment decision making, and (4) quantify and visualize the contribution of each marker in the model that drive patient-specific classifications.

## 2 Subjects and methods

### 2.1 Data source

All records were extracted from the EMR system of West China Hospital (WCH), Sichuan University. WCH is one of the largest single-site hospitals in the world and a leading medical center of West China, treating complicated and severe cases. The hospital has 4,300 beds and more than 10,000 medical staff. The psychiatric specialty of WCH provides medical services for a large number of patients with mental illness, with more than 300,000 outpatient visits and more than 6,000 discharged patients per year. As one of the most renowned medical centers in China, WCH’s clinical practice represents the current situation of patients with mental illness in China. In 2009, an EMR system integrated with the Health Information System (HIS) and the Laboratory Information System (LIS) was adopted in all departments throughout the hospital, which was set as the starting time of our data extraction.

### 2.2 Study subjects

**Figure 1:**
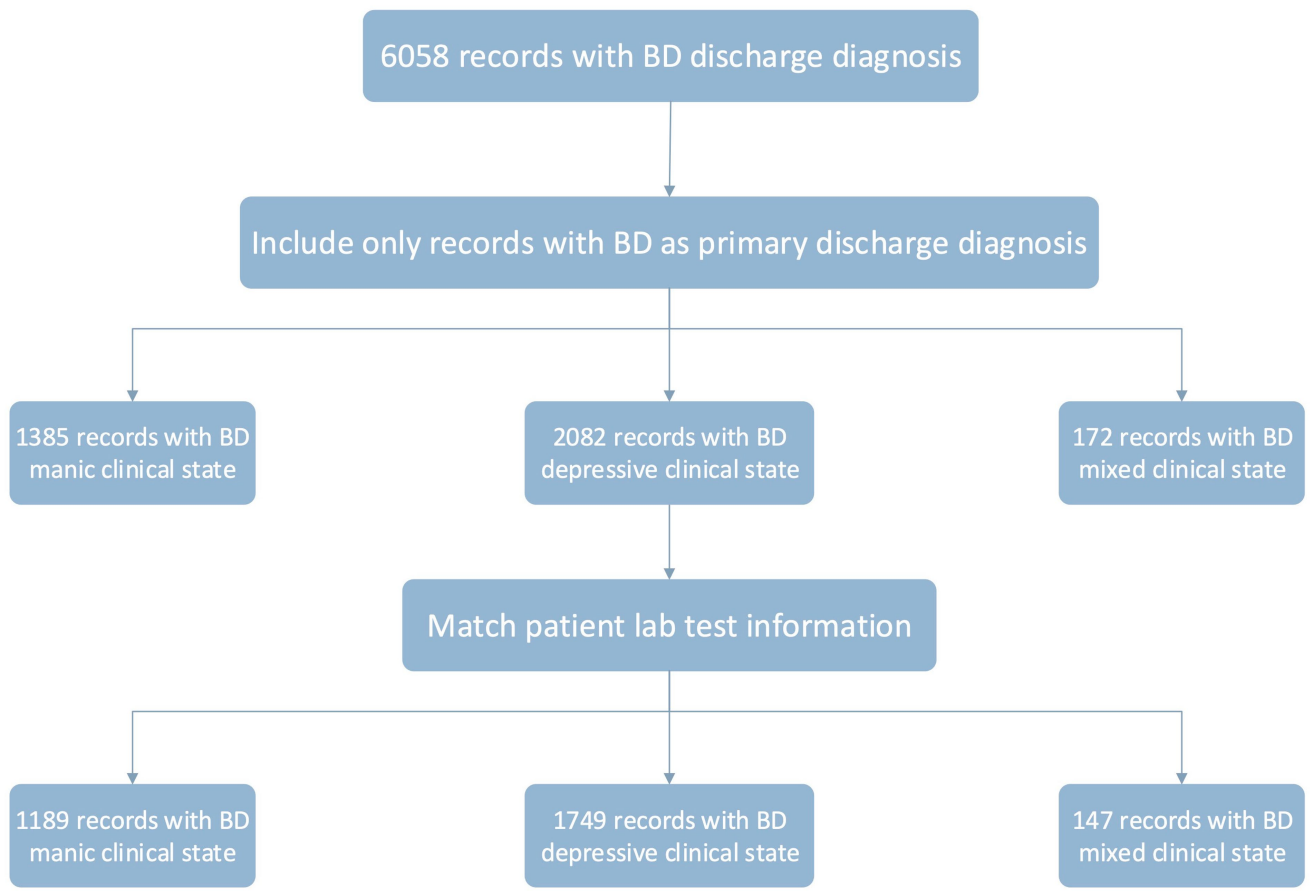
Flowchart of the study subjects.

This study included all inpatients with diagnosis as BD from all departments of WCH from January 2009 to December 2018 (10 years). Six thousand fifty-eight records of inpatients with discharged diagnosis as BD were extracted from the EMR system, identified by the International Classification of Diseases, 10th revision (ICD-10) codes. We used the presence of an F31 ICD-10 code in either the main or supplementary position to signify a BD-related admission. Four thousand seven hundred sixty-one records with a principal diagnosis of BD were kept. Based on this cohort, records were excluded if they were: (1) of patients with non-Chinese nationalities; (2) of patients with subtype diagnoses not specified; (3) of patients’ lab test information missing; (4) duplicated storage (records with the same inpatient code and case code). The data were checked for missing values, and records with any missing value, were excluded from the analysis. The remaining 1,189 records with BD manic clinical state, 1,749 records with BD depressive clinical state and 147 records with BD mixed clinical state were included in the analyses (Figure 1), yielding a total study cohort of 3,085 records.

### 2.3 Measures

#### 2.3.1 Definition of outcome

Compared with clinical diagnostic interviews, EMR-based diagnostic data can be used to identify patients with bipolar disorder and control subjects with high specificity and predictive value (27). The outcome measure in this study was defined as the main discharge diagnosis of each subject, identified by ICD-10 codes. The discharge diagnosis is the gold standard for the characterization of the diagnostic categories, which is directly evaluated by trained psychiatrists using structured or semi-structured diagnostic interviews at admission and repeatedly confirmed (or even revised) during the hospitalization.

#### 2.3.2 Features

The time course of this study spanned from each patient’s admission time to the discharge time. We extracted various categories of features from the original medical records, including sociodemographic information (age at admission, gender, marital status, job, ethnicity, source of payment, province of hometown), vital signs based on basic body check at admission (pulse, breathing, nutrition, temperature, systolic and diastolic pressure), prior illness history (allergy, blood transfusion, drug use, surgery), diagnoses, whether the patient had a diagnosis of a medical comorbidity (other mental disorders, endocrine diseases, nervous diseases, digestive diseases, circulatory diseases, respiratory diseases, and cancer), the number of comorbidity in each disease system, laboratory tests (routine blood, urine, stool and biochemical examination), and other information. Each patient might have more than once laboratory tests during the hospitalization. We extracted the first recorded laboratory test results during the inpatient stay of all subjects as the laboratory diagnostic criteria. For each laboratory test type, only the most common result types (listed by result name like platelet count, red blood cell count, absolute of lymphocyte, etc.) were used in the models. For each laboratory result type, the numerical value, or the categorical level (in the normal range or higher or lower) were included as features. No laboratory data after the first recorded date were included in analyses.

Unstructured data (chief complaint) was processed by removing all digits and punctuation. The text data was then split into words using the jieba package in R. To filter out meaningless words, we added a list of stop words and medical dictionaries. This helped to remove noise and irrelevant terms. We also prevented certain medical nouns from being split apart [details of which have been presented in our previous work (31)]. To identify the most informative words, we calculated the term frequency scores. These scores helped us to identify the words that were most representative of patients’ main symptoms and current mood states. We referred to these words as features, including “lowering of mood,” “elevation of mood,” “mood instability,” “bad sleep,” “provoke,” “worry,” “talkativeness,” “suicide ideation,” “pain,” “appetite disturbances,” “auditory hallucination,” and “relapse and worsen of symptoms.” The value of each symptom feature depended on whether the chief complaint of a patient included the above key words representing their symptoms and mood states. Specifically, a value of 1 was assigned if the patient’s chief complaint included a specific word related to their symptoms or mood states, and a value of 0 was assigned if it did not include that word. This approach helped to encode the patients’ chief complaints into numerical features for further analysis and modeling.

Overall, 198 features were recruited into the original data pool with no missing values (details in the Supplementary Information). Three groups (manic, mixed, and depressive BD) were compared regarding all included features, by using the chi-square test for categorical features and *t*-test for continuous features in the univariate-filtering step. Those with *p*-value <0.05 were considered as significant features.

### 2.4 Machine learning classification

This study was undertaken to determine the potential for multi-system composite features as predictors to discriminate between manic, depressive, and mixed clinical states of bipolar disorder patients in a data-driven approach. Only the significant features at early admission (sociodemographics, vital signs, prior illness history, comorbidities, chief complaints, and laboratory tests) were used in the ML model. All analyses were implemented using RStudio (Version 3.6.1 for Windows). In our study, four ML classifiers were selected:

#### 2.4.1 Random forests

Random forests (RF) algorithm works by modeling several decision trees which learn and make predictions independently, and outputs a combined single prediction that is same or better than the output made by the modeled decision trees (32). RF can be used for continuous and/or categorical input variables and allow class weighting to adjust for unequal sampling schemes. RF does not overfit the data, so it can be used for problems in which the number of input variables is much larger than the number of observations. It also provides measures of feature importance and proximities that can be used to interpret the data.

#### 2.4.2 Support vector machine

A support vector machine (SVM) is a supervised learning algorithm commonly used for classification and regression problems, especially in medical applications. SVM belongs to a category of ML algorithms called kernel methods, which involve transforming features using a kernel function (33). Kernel functions map the data to a different, often higher-dimensional space, with the expectation that the classes will be easier to separate after this transformation. This transformation can potentially simplify complex non-linear decision boundaries into linear ones in the higher-dimensional feature space.

#### 2.4.3 XGBoost (eXtreme Gradient Boosting)

XGBoost (XGB) is an optimized distributed gradient boosting library known for its high efficiency, flexibility, and portability (32). XGBoost’s parallel tree boosting algorithm effectively solves a wide range of data science problems with speed and precision. It was chosen as the preferred method for building diagnostic algorithms due to its ability to handle missing values, detect nonlinear relationships and interactions between variables, remain robust in the presence of correlated features, and provide interpretable results.

#### 2.4.4 Logistic regression

Logistic regression (LR) can be either binomial or multinomial. Like other forms of regression analysis, LR utilizes one or more predictor variables, which can be continuous or categorical data. The expected value of the response variable is adjusted to fit the predictors, and the regression function is a sigmoid function that transforms a real number into a value between 0 and 1. LR employs the maximum likelihood estimation method to estimate the model coefficients (34).

We used the holdout method, and randomly divided the clinical cohort into 80% training and 20% testing data, maintaining the proportion of patients in each clinical state. The training dataset was used to train the four algorithms and optimize their parameters for better classifier construction. The classifiers were then calibrated using the testing dataset, which was not utilized for model selection or parameter tuning. Fine-tuning involved adjusting the parameter combinations within the trainControl function. The parameters resulting in the best classification performance for each algorithm were chosen using 10-fold cross-validation on the training data. The learned parameters were then used to construct a model for the entire training set and to make predictions on the testing data. All the classifiers utilized in this study were fine-tuned. Several classifiers have been selected to avoid bias toward the use of a particular classifier.

Due to the unbalanced sample size in each group (see Figure 1), particularly the BD mixed episode group, we sought to balance our dataset by randomly over-sampling the positive class (BD mixed episode) in the training set to achieve a balanced ratio between the positive and negative (BD manic episode or BD depressive episode) classes. The testing set distributions were not modified to reflect the reality of class imbalance during prediction, and the reported performance reflects those raw distributions. Seven classification runs were performed with each classifier: two multiclass classifications (non-resampling and resampling) between BD manic, depressive, and mixed episodes, three non-resampling binary classifications (manic/depressive, manic/mixed, and depressive/mixed), and two resampling binary classifications (over-sampling manic/mixed and depressive/mixed).

### 2.5 Explainable classification

An important development towards enhancing the practical medical decision support provided by ML is the ability to offer straightforward explanations for predictions generated by arbitrarily intricate models, thus mitigating the typical trade-off between accuracy and interpretability. Explainable ML methods identify the specific characteristics that lead to the classification of each patient, which is crucial for determining targeted diagnostic markers and clinical prediction rules. In our work, we utilized a Shapley additive explanations (SHAP) algorithm in our classification model to obtain explanations of the features that drive patient-specific classifications. SHAP is a model-agnostic representation of feature importance where the impact of each feature on a particular prediction is characterized using Shapley values-a concept derived from cooperative game theory (35). A Shapley value signifies, given the current set of feature values, how much a single feature in the context of its interaction with other features contributes to the difference between the actual prediction and the mean prediction (36). The SHAP value for a feature reflects its compound effect when interacting with the other features. For comparison, we also showcased how specific features contribute in different classification scenarios, particularly, in three binary classifications of clinical states (manic/depressive, manic/mixed, and depressive/mixed) in BD patients.

### 2.6 Performance metrics of the ML models

To evaluate the ability to discriminate BD mixed episode, manic episode, and depressive episode, we computed the area under the receiver operating characteristics (ROC) curves (AUC). The AUC provides valuable insights into the relevant question of what proportion of true cases (sensitivity) and the proportion of false cases (specificity) the algorithm can correctly identify at different probability cutoffs. For multiclass classifications, we employed a confusion matrix as performance metric. All performance metrics were derived from the holdout testing dataset. The code is available upon request.

## 3 Results

### 3.1 Descriptive analyses

#### 3.1.1 Sociodemographic features

We included 3,085 records extracted from the EMR database, with 1,189 records representing BD manic episode (38.54%), 147 records representing BD mixed episode (4.77%) and 1,749 records as BD depressive episode (56.69%). We compared these three groups across all clinical, biological, and sociodemographic features. Table 1 provides a summary of the basic sociodemographic characteristics of the full cohort. The mean (SD) age of the study population was 36.86 (17.05) years, and 63% of patients were female. Although the three groups did not differ significantly in the ethnicity and source of patient, they did differ on gender, age, age group, job, marital status, type of payment. Notably, patients with BD mixed episode were more likely to be female (70.1% vs. 66.3% BD depressive episode and 57.2% BD manic episode; *p* < 0.001), belong to the age group of 0–17 (16.3% vs. 9.0% BD depressive episode and 14.6% BD manic episode; *p* < 0.001), and be single (53.7% vs. 32.8% BD depressive episode and 50.7% BD manic episode; *p* < 0.001).

**Table 1:** Sociodemographic and clinical characteristics of the study population.

Characteristic	All (*n* = 3,085)	BD-manic (*n* = 1,189)	BD-depressive (*n* = 1,749)	BD-mixed (*n* = 147)	*p*-value
***Sociodemographic***					
**Gender = Male (%)**	1,142 (37.0)	509 (42.8)	589 (33.7)	44 (29.9)	**<0.001**
**Age [mean (SD)]**	36.86 (17.05)	32.28 (15.45)	40.35 (17.43)	32.33 (15.04)	**<0.001**
**Age group (%)**					**<0.001**
0–17	354 (11.5)	173 (14.6)	157 (9.0)	24 (16.3)	
18–35	1,273 (41.3)	615 (51.7)	592 (33.8)	66 (44.9)	
36–60	1,097 (35.6)	314 (26.4)	734 (42.0)	49 (33.3)	
61-	361 (11.7)	87 (7.3)	266 (15.2)	8 (5.4)	
**Job status (%)**					**<0.001**
Basic	419 (13.6)	150 (12.6)	252 (14.4)	17 (11.6)	
Freelance	154 (5.0)	79 (6.6)	67 (3.8)	8 (5.4)	
Labor	428 (13.9)	168 (14.1)	236 (13.5)	24 (16.3)	
Management	111 (3.6)	36 (3.0)	72 (4.1)	3 (2.0)	
Other	376 (12.2)	162 (13.6)	193 (11.0)	21 (14.3)	
Retired	381 (12.4)	79 (6.6)	288 (16.5)	14 (9.5)	
Service	158 (5.1)	40 (3.4)	113 (6.5)	5 (3.4)	
Student	720 (23.3)	338 (28.4)	341 (19.5)	41 (27.9)	
Unemployed	338 (11.0)	137 (11.5)	187 (10.7)	14 (9.5)	
**Marital status (%)**					**<0.001**
Divorced	162 (5.3)	61 (5.1)	98 (5.6)	3 (2.0)	
Married	1,554 (50.4)	496 (41.7)	994 (56.8)	64 (43.5)	
Single	1,255 (40.7)	603 (50.7)	573 (32.8)	79 (53.7)	
Unknown	10 (0.3)	5 (0.4)	5 (0.3)	0 (0.0)	
Widow	104 (3.4)	24 (2.0)	79 (4.5)	1 (0.7)	
**Ethnicity (%)**					**0.697**
Han	2,866 (92.9)	1,107 (93.1)	1,622 (92.7)	137 (93.2)	
Other	78 (2.5)	25 (2.1)	48 (2.7)	5 (3.4)	
Zang	141 (4.6)	57 (4.8)	79 (4.5)	5 (3.4)	
**Payment type (%)**					**<0.001**
Cash	1,728 (56.0)	717 (60.3)	926 (52.9)	85 (57.8)	
City coverage	1,108 (35.9)	404 (34.0)	658 (37.6)	46 (31.3)	
Province coverage	248 (8.0)	67 (5.6)	165 (9.4)	16 (10.9)	
Special	1 (0.0)	1 (0.1)	0 (0.0)	0 (0.0)	
**Source of patient (%)**					**0.144**
City	1,656 (53.7)	622 (52.3)	962 (55.0)	72 (49.0)	
In province	1,020 (33.1)	421 (35.4)	543 (31.0)	56 (38.1)	
Out province	356 (11.5)	125 (10.5)	213 (12.2)	18 (12.2)	
Unknown	53 (1.7)	21 (1.8)	31 (1.8)	1 (0.7)	
***Symptom***					
**Lowering of mood = TRUE (%)**	2,398 (77.7)	729 (61.3)	1,566 (89.5)	103 (70.1)	**<0.001**
**Elevation of mood = TRUE (%)**	1,672 (54.2)	834 (70.1)	771 (44.1)	67 (45.6)	**<0.001**
**Mood instability = TRUE (%)**	2,088 (67.7)	760 (63.9)	1,228 (70.2)	100 (68.0)	**0.002**
**Bad sleep = TRUE (%)**	723 (23.4)	208 (17.5)	489 (28.0)	26 (17.7)	**<0.001**
**Provoke = TRUE (%)**	495 (16.0)	316 (26.6)	147 (8.4)	32 (21.8)	**<0.001**
**Worry = TRUE (%)**	455 (14.7)	129 (10.8)	310 (17.7)	16 (10.9)	**<0.001**
**Talkativeness = TRUE (%)**	816 (26.5)	603 (50.7)	180 (10.3)	33 (22.4)	**<0.001**
**Suicide ideation = TRUE (%)**	161 (5.2)	30 (2.5)	118 (6.7)	13 (8.8)	**<0.001**
**Pain = TRUE (%)**	175 (5.7)	23 (1.9)	143 (8.2)	9 (6.1)	**<0.001**
**Appetite disturbances = TRUE (%)**	37 (1.2)	4 (0.3)	29 (1.7)	4 (2.7)	**0.001**
**Auditory hallucination = TRUE (%)**	72 (2.3)	30 (2.5)	40 (2.3)	2 (1.4)	**0.665**
**Recurrence of symptoms = TRUE (%)**	1,144 (37.1)	372 (31.3)	736 (42.1)	36 (24.5)	**<0.001**
**Worsen of symptoms = TRUE (%)**	774 (25.1)	228 (19.2)	503 (28.8)	43 (29.3)	**<0.001**
***Vital signs***					
**Temperature [mean (SD)]**	36.57 (0.26)	36.59 (0.27)	36.56 (0.25)	36.55 (0.23)	**0.002**
**Pulse [mean (SD)]**	85.13 (13.98)	88.63 (14.54)	82.96 (13.28)	82.67 (11.58)	**<0.001**
**Breathing [mean (SD)]**	19.78 (0.75)	19.79 (0.70)	19.79 (0.78)	19.67 (0.70)	**0.152**
**Systolic blood pressure [mean (SD)]**	120.97 (14.96)	122.26 (13.96)	120.18 (15.47)	119.90 (15.90)	**0.001**
**Diastolic blood pressure [mean (SD)]**	77.87 (11.25)	78.77 (11.49)	77.18 (11.12)	78.64 (10.26)	**0.001**
**Nutrition (%)**					**0.656**
Bad	13 (0.4)	3 (0.3)	9 (0.5)	1 (0.7)	
Good	3,001 (97.3)	1,161 (97.6)	1,699 (97.1)	141 (95.9)	
Medium	71 (2.3)	25 (2.1)	41 (2.3)	5 (3.4)	
**Cooperation = TRUE (%)**	2,941 (95.3)	1,073 (90.2)	1,729 (98.9)	139 (94.6)	**<0.001**
***Prior illness history***					
**History of surgery = YES (%)**	899 (29.1)	277 (23.3)	585 (33.4)	37 (25.2)	**<0.001**
**History of allergy = YES (%)**	384 (12.4)	111 (9.3)	254 (14.5)	19 (12.9)	**<0.001**
**History of blood transfusion = YES (%)**	61 (2.0)	14 (1.2)	46 (2.6)	1 (0.7)	**0.011**
**History of drug use (%)**					**<0.001**
None	2,049 (66.4)	828 (69.6)	1,118 (63.9)	103 (70.1)	
Often	738 (23.9)	234 (19.7)	475 (27.2)	29 (19.7)	
Sometimes	173 (5.6)	78 (6.6)	86 (4.9)	9 (6.1)	
Unknown	125 (4.1)	49 (4.1)	70 (4.0)	6 (4.1)	
**History reliability (%)**					**0.039**
Almost reliable	310 (10.0)	142 (11.9)	151 (8.6)	17 (11.6)	
Reliable	2,770 (89.8)	1,044 (87.8)	1,596 (91.3)	130 (88.4)	
Unreliable	5 (0.2)	3 (0.3)	2 (0.1)	0 (0.0)	
***Medical comorbidity***					
**Comorbidity number [mean (SD)]**	0.90 (1.41)	0.62 (1.20)	1.10 (1.52)	0.75 (1.21)	**<0.001**
**Psychiatric comorbidity = YES (%)**	300 (9.7)	48 (4.0)	236 (13.5)	16 (10.9)	**<0.001**
**Psychiatric comorbidity number [mean (SD)]**	0.10 (0.33)	0.04 (0.21)	0.14 (0.38)	0.12 (0.34)	**<0.001**
**Endocrine comorbidity = YES (%)**	454 (14.7)	135 (11.4)	302 (17.3)	17 (11.6)	**<0.001**
**Endocrine comorbidity number [mean (SD)]**	0.18 (0.48)	0.14 (0.44)	0.21 (0.50)	0.14 (0.44)	**0.001**
**Nervous comorbidity = YES (%)**	104 (3.4)	25 (2.1)	73 (4.2)	6 (4.1)	**0.008**
**Nervous comorbidity number [mean (SD)]**	0.04 (0.24)	0.03 (0.19)	0.05 (0.26)	0.06 (0.31)	**0.015**
**Digestive comorbidity = YES (%)**	304 (9.9)	67 (5.6)	225 (12.9)	12 (8.2)	**<0.001**
**Digestive comorbidity number [mean (SD)]**	0.12 (0.42)	0.07 (0.33)	0.16 (0.47)	0.10 (0.34)	**<0.001**
**Circulatory comorbidity = YES (%)**	314 (10.2)	69 (5.8)	233 (13.3)	12 (8.2)	**<0.001**
**Circulatory comorbidity number [mean (SD)]**	0.13 (0.41)	0.07 (0.33)	0.17 (0.46)	0.08 (0.27)	**<0.001**
**Respiratory comorbidity = YES (%)**	156 (5.1)	68 (5.7)	81 (4.6)	7 (4.8)	**0.412**
**Respiratory comorbidity number [mean (SD)]**	0.06 (0.25)	0.06 (0.26)	0.05 (0.25)	0.05 (0.26)	**0.653**
**Cancer comorbidity = YES (%)**	11 (0.4)	5 (0.4)	6 (0.3)	0 (0.0)	**0.715**
**Cancer comorbidity number [mean (SD)]**	0.00 (0.07)	0.00 (0.06)	0.00 (0.07)	0.00 (0.00)	**0.77**

The bold values presented in Table 1 indicate the features of patients, as well as the p-value results obtained from hypothesis tests.

#### 3.1.2 Symptoms

Patients experiencing depressive episodes were more likely to exhibit symptoms such as lowering of mood, mood instability, poor sleep, worry, pain and relapse of these symptoms. Patients in the manic episode group were more likely to have symptoms like elevation of mood, irritability, and talkativeness. The mixed episode group was characterized by symptoms like suicide ideation, appetite disturbances, and an exacerbation of the aforementioned symptoms. It is noteworthy that the proportion of symptoms in patients with mixed episodes falls somewhere between that of patients with manic and depressive episodes. Three groups only did not differ significantly in terms of auditory hallucination. This finding supports our initial hypothesis that unstructured data extracted from the chief complaint is a crucial tool for assessing patients’ symptoms and current mood states in order to discriminate between the manic and depressive clinical states of BD patients. While mixed states are notoriously difficult to identify due to their composite symptoms of depression and mania, these data underscore the clinical importance of assessing patients with bipolar disorder for current symptoms of both poles of the illness regardless of their self-reported current mood state (Table 1).

#### 3.1.3 Vital signs, prior illness history and comorbidity

Table 1 summarizes the characteristics of vital signs based on basic body checks prior to hospitalization. Compared to patients experiencing manic and depressive episodes, those in mixed episodes were more likely to exhibit lower mean temperatures, slower pulse rates, and lower systolic blood pressures. Patients in manic episodes, on the other hand, displayed the highest mean values for temperature, pulse, systolic, and diastolic blood pressures compared to the other two groups. In terms of prior history, patients in depressive episodes were more likely to have a history of allergies and surgery, and they also had slightly higher probabilities of a history of drug use and blood transfusion. Table 1 also outlines the cross-sectional rate of psychiatric and other comorbidity conditions in different clinical states of BD. Notably, the depression group had a significantly higher comorbidity rate than the other two groups, including higher rates of psychiatric comorbidity conditions, endocrine comorbidity conditions, digestive comorbidity conditions, circulatory comorbidity conditions, and slightly higher rates of nervous comorbidity conditions. Endocrine diseases were the most prevalent comorbid conditions in BD patients, followed by circulatory diseases.

#### 3.1.4 Laboratory findings

Table 2 presents the results of laboratory examination dataset analysis in the study population. The dataset includes routine blood, biochemical, urine, and stool examination results obtained before hospitalization.

**Table 2:** Routine laboratory test results of the study population.

Characteristic	All (*n* = 3,085)	BD-manic (*n* = 1,189)	BD-depressive (*n* = 1,749)	BD-mixed (*n* = 147)	*p*-value
***Routine blood test findings***					
**Red cell distribution width (RDW) CV value [mean (SD)]**	13.47 (1.27)	13.51 (1.32)	13.45 (1.22)	13.45 (1.36)	**0.487**
**RDW CV level (%)**					**0.781**
High	381 (12.4)	157 (13.2)	205 (11.7)	19 (12.9)	
Low	13 (0.4)	5 (0.4)	7 (0.4)	1 (0.7)	
Normal	2,691 (87.2)	1,027 (86.4)	1,537 (87.9)	127 (86.4)	
**Red blood cell (RBC) SD value [mean (SD)]**	44.69 (4.06)	44.57 (4.26)	44.80 (3.94)	44.22 (3.90)	**0.112**
**RBC SD level (%)**					**0.07**
High	68 (2.2)	32 (2.7)	34 (1.9)	2 (1.4)	
Low	52 (1.7)	22 (1.9)	24 (1.4)	6 (4.1)	
Normal	2,965 (96.1)	1,135 (95.5)	1,691 (96.7)	139 (94.6)	
**White blood cell count (WCC) value (mean (SD))**	6.41 (2.19)	6.95 (2.57)	6.06 (1.84)	6.19 (1.75)	**<0.001**
**WCC level (%)**					**<0.001**
High	221 (7.2)	135 (11.4)	79 (4.5)	7 (4.8)	
Low	107 (3.5)	32 (2.7)	67 (3.8)	8 (5.4)	
Normal	2,757 (89.4)	1,022 (86.0)	1,603 (91.7)	132 (89.8)	
**Percentage of monocyte (POM) value [mean (SD)]**	6.60 (1.90)	6.71 (1.94)	6.50 (1.87)	6.92 (1.79)	**0.002**
**POM level (%)**					**0.006**
High	193 (6.3)	96 (8.1)	87 (5.0)	10 (6.8)	
Low	32 (1.0)	10 (0.8)	22 (1.3)	0 (0.0)	
Normal	2,860 (92.7)	1,083 (91.1)	1,640 (93.8)	137 (93.2)	
**Absolute of monocyte (AOM) value [mean (SD)]**	0.42 (0.17)	0.46 (0.19)	0.39 (0.15)	0.43 (0.17)	**<0.001**
**AOM level (%)**					**<0.001**
High	316 (10.2)	167 (14.0)	134 (7.7)	15 (10.2)	
Low	6 (0.2)	1 (0.1)	5 (0.3)	0 (0.0)	
Normal	2,763 (89.6)	1,021 (85.9)	1,610 (92.1)	132 (89.8)	
**RBC count (RBCC) value [mean (SD)]**	4.52 (0.56)	4.57 (0.56)	4.48 (0.55)	4.50 (0.55)	**<0.001**
**RBCC level (%)**					**0.409**
High	115 (3.7)	46 (3.9)	61 (3.5)	8 (5.4)	
Low	300 (9.7)	103 (8.7)	182 (10.4)	15 (10.2)	
Normal	2,670 (86.5)	1,040 (87.5)	1,506 (86.1)	124 (84.4)	
**Hematocrit value [mean (SD)]**	0.42 (0.05)	0.42 (0.05)	0.42 (0.05)	0.41 (0.05)	**0.2**
**Hematocrit level (%)**					**0.608**
High	107 (3.5)	38 (3.2)	65 (3.7)	4 (2.7)	
Low	318 (10.3)	131 (11.0)	169 (9.7)	18 (12.2)	
Normal	2,660 (86.2)	1,020 (85.8)	1,515 (86.6)	125 (85.0)	
**Percentage of lymphocyte (POL) value [mean (SD)]**	31.42 (9.75)	29.86 (10.29)	32.34 (9.30)	32.97 (8.89)	**<0.001**
**POL level (%)**					**<0.001**
High	140 (4.5)	55 (4.6)	81 (4.6)	4 (2.7)	
Low	380 (12.3)	207 (17.4)	156 (8.9)	17 (11.6)	
Normal	2,565 (83.1)	927 (78.0)	1,512 (86.4)	126 (85.7)	
**Absolute of lymphocyte (AOL) value [mean (SD)]**	1.92 (0.64)	1.95 (0.67)	1.90 (0.62)	1.98 (0.58)	**0.068**
**AOL level (%)**					**0.931**
High	98 (3.2)	40 (3.4)	55 (3.1)	3 (2.0)	
Low	215 (7.0)	84 (7.1)	120 (6.9)	11 (7.5)	
Normal	2,772 (89.9)	1,065 (89.6)	1,574 (90.0)	133 (90.5)	
**Average red blood cell (ARBC) HGB value [mean (SD)]**	29.97 (2.49)	29.94 (2.36)	30.02 (2.52)	29.56 (3.11)	**0.087**
**ARBC HGB level (%)**					**0.217**
High	73 (2.4)	27 (2.3)	40 (2.3)	6 (4.1)	
Low	193 (6.3)	64 (5.4)	116 (6.6)	13 (8.8)	
Normal	2,819 (91.4)	1,098 (92.3)	1,593 (91.1)	128 (87.1)	
**ARBC HGB concentration value [mean (SD)]**	327.76 (11.54)	328.97 (11.77)	327.18 (11.41)	324.88 (10.17)	**<0.001**
**ARBC HGB concentration level (%)**					**0.188**
High	27 (0.9)	15 (1.3)	11 (0.6)	1 (0.7)	
Low	360 (11.7)	130 (10.9)	207 (11.8)	23 (15.6)	
Normal	2,698 (87.5)	1,044 (87.8)	1,531 (87.5)	123 (83.7)	
**Average red blood cell volume (ARBCV) value [mean (SD)]**	91.40 (6.56)	91.01 (6.44)	91.70 (6.45)	90.89 (8.32)	**0.011**
**ARBCV level (%)**					**0.36**
High	116 (3.8)	43 (3.6)	66 (3.8)	7 (4.8)	
Low	153 (5.0)	50 (4.2)	92 (5.3)	11 (7.5)	
Normal	2,816 (91.3)	1,096 (92.2)	1,591 (91.0)	129 (87.8)	
**Percentage of basophil (POB) value [mean (SD)]**	0.45 (0.29)	0.41 (0.28)	0.48 (0.30)	0.47 (0.28)	**<0.001**
**POB level = normal (%)**	2,973 (96.4)	1,155 (97.1)	1,676 (95.8)	142 (96.6)	**0.172**
**Percentage of eosinophil (POE) value [mean (SD)]**	2.34 (1.92)	2.24 (1.87)	2.40 (1.94)	2.39 (1.98)	**0.068**
**POE level (%)**					**0.037**
High	79 (2.6)	30 (2.5)	45 (2.6)	4 (2.7)	
Low	192 (6.2)	94 (7.9)	93 (5.3)	5 (3.4)	
Normal	2,814 (91.2)	1,065 (89.6)	1,611 (92.1)	138 (93.9)	
**Hemoglobin value [mean (SD)]**	134.70 (16.50)	136.33 (16.50)	133.81 (16.52)	132.08 (15.21)	**<0.001**
**Hemoglobin level (%)**					**0.526**
High	67 (2.2)	25 (2.1)	40 (2.3)	2 (1.4)	
Low	362 (11.7)	127 (10.7)	214 (12.2)	21 (14.3)	
Normal	2,656 (86.1)	1,037 (87.2)	1,495 (85.5)	124 (84.4)	
**Platelet count (PC) value [mean (SD)]**	203.40 (67.28)	209.08 (68.94)	199.81 (66.78)	200.10 (55.78)	**0.001**
**PC level (%)**					**0.029**
High	239 (7.7)	104 (8.7)	129 (7.4)	6 (4.1)	
Low	136 (4.4)	43 (3.6)	90 (5.1)	3 (2.0)	
Normal	2,710 (87.8)	1,042 (87.6)	1,530 (87.5)	138 (93.9)	
**Percentage of neutrophil (PON) value [mean (SD)]**	59.17 (10.75)	60.77 (11.38)	58.25 (10.27)	57.26 (9.37)	**<0.001**
**PON level (%)**					**<0.001**
High	262 (8.5)	145 (12.2)	112 (6.4)	5 (3.4)	
Low	143 (4.6)	58 (4.9)	80 (4.6)	5 (3.4)	
Normal	2,680 (86.9)	986 (82.9)	1,557 (89.0)	137 (93.2)	
**Absolute of neutrophil (AON) value [mean (SD)]**	3.91 (1.93)	4.38 (2.33)	3.61 (1.56)	3.62 (1.43)	**<0.001**
**AON level (%)**					**<0.001**
High	259 (8.4)	157 (13.2)	93 (5.3)	9 (6.1)	
Low	145 (4.7)	43 (3.6)	94 (5.4)	8 (5.4)	
Normal	2,681 (86.9)	989 (83.2)	1,562 (89.3)	130 (88.4)	
***Routine biochemical test findings***					
**Alanine aminotransferase (ALA) value [mean (SD)]**	24.48 (27.65)	25.93 (25.72)	23.66 (29.08)	22.54 (24.59)	**0.063**
**ALA level (%)**					**0.168**
High	300 (9.7)	133 (11.2)	158 (9.0)	9 (6.1)	
Low	6 (0.2)	2 (0.2)	4 (0.2)	0 (0.0)	
Normal	2,779 (90.1)	1,054 (88.6)	1,587 (90.7)	138 (93.9)	
**Aspartate aminotransferase (ASA) value [mean (SD)]**	23.84 (18.64)	26.06 (16.90)	22.45 (19.42)	22.52 (20.87)	**<0.001**
**ASA level (%)**					**<0.001**
High	264 (8.6)	149 (12.5)	105 (6.0)	10 (6.8)	
Low	1 (0.0)	0 (0.0)	1 (0.1)	0 (0.0)	
Normal	2,820 (91.4)	1,040 (87.5)	1,643 (93.9)	137 (93.2)	
**Creatine kinase (CK) value [mean (SD)]**	155.02 (382.12)	241.27 (510.16)	99.95 (267.43)	112.51 (107.73)	**<0.001**
**CK level (%)**					**<0.001**
High	473 (15.3)	347 (29.2)	106 (6.1)	20 (13.6)	
Low	2 (0.1)	1 (0.1)	1 (0.1)	0 (0.0)	
Normal	2,610 (84.6)	841 (70.7)	1,642 (93.9)	127 (86.4)	
**Lactate dehydrogenase (LD) value [mean (SD)]**	167.07 (51.30)	186.23 (61.08)	154.82 (39.89)	157.83 (36.15)	**<0.001**
**LD level (%)**					**<0.001**
High	285 (9.2)	194 (16.3)	82 (4.7)	9 (6.1)	
Low	185 (6.0)	39 (3.3)	132 (7.5)	14 (9.5)	
Normal	2,615 (84.8)	956 (80.4)	1,535 (87.8)	124 (84.4)	
**Urea value [mean (SD)]**	4.63 (1.55)	4.69 (1.66)	4.60 (1.48)	4.48 (1.43)	**0.11**
**Urea level (%)**					**0.015**
High	88 (2.9)	48 (4.0)	37 (2.1)	3 (2.0)	
Low	328 (10.6)	138 (11.6)	175 (10.0)	15 (10.2)	
Normal	2,669 (86.5)	1,003 (84.4)	1,537 (87.9)	129 (87.8)	
**Total bilirubin (TB) value [mean (SD)]**	10.27 (5.77)	10.40 (6.55)	10.16 (5.14)	10.43 (6.18)	**0.498**
**TB level (%)**					**0.023**
High	40 (1.3)	22 (1.9)	16 (0.9)	2 (1.4)	
Low	289 (9.4)	129 (10.8)	144 (8.2)	16 (10.9)	
Normal	2,756 (89.3)	1,038 (87.3)	1,589 (90.9)	129 (87.8)	
**Direct bilirubin (DB) value [mean (SD)]**	3.73 (2.92)	4.04 (4.03)	3.52 (1.87)	3.71 (2.25)	**<0.001**
**DB level (%)**					**0.001**
High	74 (2.4)	45 (3.8)	24 (1.4)	5 (3.4)	
Low	6 (0.2)	1 (0.1)	5 (0.3)	0 (0.0)	
Normal	3,005 (97.4)	1,143 (96.1)	1,720 (98.3)	142 (96.6)	
**Indirect bilirubin (IDB) value [mean (SD)]**	6.54 (3.69)	6.37 (3.77)	6.64 (3.60)	6.73 (4.10)	**0.123**
**IDB level = normal (%)**	3,058 (99.1)	1,177 (99.0)	1,736 (99.3)	145 (98.6)	**0.608**
**Total protein (TP) value [mean (SD)]**	68.42 (5.57)	69.79 (5.75)	67.50 (5.31)	68.24 (4.80)	**<0.001**
**TP level (%)**					**<0.001**
High	12 (0.4)	8 (0.7)	4 (0.2)	0 (0.0)	
Low	773 (25.1)	216 (18.2)	523 (29.9)	34 (23.1)	
Normal	2,300 (74.6)	965 (81.2)	1,222 (69.9)	113 (76.9)	
**Albumin value [mean (SD)]**	43.27 (4.10)	44.50 (4.29)	42.41 (3.78)	43.66 (3.52)	**<0.001**
**Albumin level (%)**					**<0.001**
High	7 (0.2)	4 (0.3)	3 (0.2)	0 (0.0)	
Low	576 (18.7)	161 (13.5)	399 (22.8)	16 (10.9)	
Normal	2,502 (81.1)	1,024 (86.1)	1,347 (77.0)	131 (89.1)	
**Creatinine value [mean (SD)]**	65.58 (16.49)	66.56 (16.13)	65.09 (15.77)	63.39 (25.12)	**0.016**
**Creatinine level (%)**					**0.15**
High	9 (0.3)	5 (0.4)	3 (0.2)	1 (0.7)	
Low	46 (1.5)	19 (1.6)	22 (1.3)	5 (3.4)	
Normal	3,030 (98.2)	1,165 (98.0)	1,724 (98.6)	141 (95.9)	
**Glucose value [mean (SD)]**	5.22 (1.43)	5.40 (1.63)	5.12 (1.27)	4.95 (1.30)	**<0.001**
**Glucose level (%)**					**<0.001**
High	502 (16.3)	253 (21.3)	233 (13.3)	16 (10.9)	
Low	95 (3.1)	38 (3.2)	49 (2.8)	8 (5.4)	
Normal	2,488 (80.6)	898 (75.5)	1,467 (83.9)	123 (83.7)	
**Alkaline phosphatase (AP) value [mean (SD)]**	75.98 (33.55)	80.93 (37.82)	72.72 (30.01)	74.67 (32.13)	**<0.001**
**AP level (%)**					**0.013**
High	90 (2.9)	50 (4.2)	38 (2.2)	2 (1.4)	
Low	274 (8.9)	97 (8.2)	162 (9.3)	15 (10.2)	
Normal	2,721 (88.2)	1,042 (87.6)	1,549 (88.6)	130 (88.4)	
**Glutamyl transpeptidase (GT) value [mean (SD)]**	28.60 (78.22)	27.07 (55.90)	28.74 (75.01)	39.40 (190.58)	**0.195**
**GT level (%)**					**0.315**
High	268 (8.7)	96 (8.1)	160 (9.1)	12 (8.2)	
Low	4 (0.1)	1 (0.1)	2 (0.1)	1 (0.7)	
Normal	2,813 (91.2)	1,092 (91.8)	1,587 (90.7)	134 (91.2)	
**Sodium value [mean (SD)]**	141.56 (2.62)	141.26 (2.61)	141.75 (2.64)	141.67 (2.35)	**<0.001**
**Sodium level (%)**					**0.29**
High	64 (2.1)	20 (1.7)	41 (2.3)	3 (2.0)	
Low	101 (3.3)	48 (4.0)	49 (2.8)	4 (2.7)	
Normal	2,920 (94.7)	1,121 (94.3)	1,659 (94.9)	140 (95.2)	
**Potassium value [mean (SD)]**	3.97 (0.36)	3.96 (0.36)	3.98 (0.37)	4.00 (0.32)	**0.355**
**Potassium level (%)**					**0.768**
High	6 (0.2)	2 (0.2)	4 (0.2)	0 (0.0)	
Low	271 (8.8)	113 (9.5)	147 (8.4)	11 (7.5)	
Normal	2,808 (91.0)	1,074 (90.3)	1,598 (91.4)	136 (92.5)	
**Chlorine value [mean (SD)]**	104.32 (3.36)	104.10 (3.43)	104.47 (3.33)	104.27 (3.10)	**0.011**
**Chlorine level (%)**					**0.031**
High	125 (4.1)	39 (3.3)	84 (4.8)	2 (1.4)	
Low	162 (5.3)	74 (6.2)	82 (4.7)	6 (4.1)	
Normal	2,798 (90.7)	1,076 (90.5)	1,583 (90.5)	139 (94.6)	
**Globulin value [mean (SD)]**	25.15 (3.80)	25.30 (3.80)	25.09 (3.82)	24.58 (3.45)	**0.057**
**Globulin level (%)**					**0.429**
High	11 (0.4)	6 (0.5)	5 (0.3)	0 (0.0)	
Low	211 (6.8)	71 (6.0)	130 (7.4)	10 (6.8)	
Normal	2,863 (92.8)	1,112 (93.5)	1,614 (92.3)	137 (93.2)	
**White ball ratio (WBR) value [mean (SD)]**	1.76 (0.32)	1.80 (0.32)	1.73 (0.31)	1.82 (0.34)	**<0.001**
**WBR level (%)**					**0.061**
High	99 (3.2)	47 (4.0)	44 (2.5)	8 (5.4)	
Low	70 (2.3)	26 (2.2)	43 (2.5)	1 (0.7)	
Normal	2,916 (94.5)	1,116 (93.9)	1,662 (95.0)	138 (93.9)	
**Uric acid (UA) value [mean (SD)]**	321.40 (96.68)	345.40 (101.77)	305.61 (90.35)	315.29 (87.77)	**<0.001**
**UA level (%)**					**<0.001**
High	370 (12.0)	199 (16.7)	155 (8.9)	16 (10.9)	
Low	111 (3.6)	25 (2.1)	80 (4.6)	6 (4.1)	
Normal	2,604 (84.4)	965 (81.2)	1,514 (86.6)	125 (85.0)	
**Hydroxybutyrate dehydrogenase (HD) [mean (SD)]**	132.63 (42.35)	148.52 (50.82)	122.43 (32.51)	125.39 (29.41)	**<0.001**
**HD level (%)**					**<0.001**
High	252 (8.2)	184 (15.5)	61 (3.5)	7 (4.8)	
Low	7 (0.2)	1 (0.1)	6 (0.3)	0 (0.0)	
Normal	2,826 (91.6)	1,004 (84.4)	1,682 (96.2)	140 (95.2)	
**Cholesterol value [mean (SD)]**	4.22 (0.97)	4.02 (0.94)	4.35 (0.97)	4.19 (0.94)	**<0.001**
**Cholesterol level (%)**					**<0.001**
High	211 (6.8)	54 (4.5)	150 (8.6)	7 (4.8)	
Low	143 (4.6)	77 (6.5)	60 (3.4)	6 (4.1)	
Normal	2,731 (88.5)	1,058 (89.0)	1,539 (88.0)	134 (91.2)	
**High density lipoprotein (HDL) value [mean (SD)]**	1.37 (0.38)	1.40 (0.40)	1.35 (0.37)	1.44 (0.36)	**0.001**
**HDL level = normal (%)**	2,846 (92.3)	1,101 (92.6)	1,605 (91.8)	140 (95.2)	**0.271**
**Low density lipoprotein (LDL) value [mean (SD)]**	2.35 (0.78)	2.18 (0.75)	2.48 (0.78)	2.28 (0.69)	**<0.001**
**LDL level = normal (%)**	2,993 (97.0)	1,164 (97.9)	1,686 (96.4)	143 (97.3)	**0.063**
**Cystatin C value [mean (SD)]**	0.85 (0.17)	0.85 (0.16)	0.86 (0.16)	0.83 (0.27)	**0.184**
**Cystatin C level (%)**					**0.67**
High	193 (6.3)	75 (6.3)	113 (6.5)	5 (3.4)	
Low	2 (0.1)	1 (0.1)	1 (0.1)	0 (0.0)	
Normal	2,890 (93.7)	1,113 (93.6)	1,635 (93.5)	142 (96.6)	
**Calcium value [mean (SD)]**	2.26 (0.12)	2.28 (0.12)	2.25 (0.12)	2.26 (0.11)	**<0.001**
**Calcium level (%)**					**0.009**
High	5 (0.2)	4 (0.3)	1 (0.1)	0 (0.0)	
Low	232 (7.5)	67 (5.6)	153 (8.7)	12 (8.2)	
Normal	2,848 (92.3)	1,118 (94.0)	1,595 (91.2)	135 (91.8)	
**Magnesium value [mean (SD)]**	0.88 (0.09)	0.89 (0.10)	0.88 (0.09)	0.88 (0.10)	**0.676**
**Magnesium level (%)**					**0.137**
High	129 (4.2)	58 (4.9)	67 (3.8)	4 (2.7)	
Low	24 (0.8)	6 (0.5)	15 (0.9)	3 (2.0)	
Normal	2,932 (95.0)	1,125 (94.6)	1,667 (95.3)	140 (95.2)	
**Serum inorganic phosphorous (SIP) value [mean (SD)]**	1.18 (0.22)	1.17 (0.23)	1.18 (0.22)	1.24 (0.21)	**0.002**
**SIP level (%)**					**0.182**
High	242 (7.8)	86 (7.2)	144 (8.2)	12 (8.2)	
Low	265 (8.6)	120 (10.1)	133 (7.6)	12 (8.2)	
Normal	2,578 (83.6)	983 (82.7)	1,472 (84.2)	123 (83.7)	
**Carbon dioxide combining power (CO**_**2**_ **CP) value [mean (SD)]**	23.98 (3.14)	23.24 (3.11)	24.50 (3.06)	23.85 (3.09)	**<0.001**
**CO**_**2**_ **CP level (%)**					**<0.001**
High	286 (9.3)	57 (4.8)	215 (12.3)	14 (9.5)	
Low	82 (2.7)	54 (4.5)	24 (1.4)	4 (2.7)	
Normal	2,717 (88.1)	1,078 (90.7)	1,510 (86.3)	129 (87.8)	
**Anion gap (AG) value [mean (SD)]**	17.24 (3.91)	17.91 (3.93)	16.75 (3.82)	17.56 (4.06)	**<0.001**
**AG level (%)**					**<0.001**
High	670 (21.7)	323 (27.2)	315 (18.0)	32 (21.8)	
Low	241 (7.8)	59 (5.0)	172 (9.8)	10 (6.8)	
Normal	2,174 (70.5)	807 (67.9)	1,262 (72.2)	105 (71.4)	

The bold values presented in Table 2 indicate the features of patients, as well as the p-value results obtained from hypothesis tests.

The results of the routine blood examination (Table 2) show that patients in manic episodes exhibit higher mean values for white blood cell count (WCC) and a higher proportion of patients with high abnormal WCC levels. In contrast, the mixed episode group displays a higher proportion of patients with low abnormal WCC levels. Additionally, patients in manic episodes have a higher proportion of patients with high abnormal monocyte (POM) levels, while those in depressive episodes show a higher proportion of patients with low abnormal POM levels. Patients in manic episodes also exhibit higher mean values for absolute of monocyte (AOM), red blood cell count (RBCC), average red blood cell (ARBC) HGB concentration, hemoglobin, platelet count (PC), percentage and absolute of neutrophil (PON, AON). Patients in mixed episodes display higher mean values for POM and percentage of lymphocyte (POL), while those in depressive episodes show higher mean values for percentage of basophil (POB).

The routine biochemical examination results (Table 2) indicate that patients in manic episodes exhibit higher mean values for aspartate aminotransferase (ASA), creatine kinase (CK), lactate dehydrogenase (LD), direct bilirubin (DB), total protein (TP), albumin, creatinine, glucose, alkaline phosphatase (AP), uric acid (UA), hydroxybutyrate dehydrogenase (HD), calcium and anion gap (AG). Statistical comparison between the three groups in the test results of routine examinations of urine and stool showed no significant difference in all indicators.

### 3.2 ML models to distinguish between BD manic, depressive, and mixed clinical states

#### 3.2.1 Classifiers

When distinguishing between manic, depressive, and mixed clinical states in BD patients upon admission, the performance of four machine learning algorithms (LR, SVM, RF, Xgboost) were evaluated for both in the multiclass classification (Table 3) and binary classification (Table 4).

**Table 3:** Multi-class classification performance of four algorithms.

Non-resampling of traindata	Confusion matrix of testdata	BD-Manic (*n* = 350)	BD-depressive (*n* = 238)	BD-Mixed (*n* = 29)	Accuracy	AUC
	***SVM***				**0.752**	**0.741**
	BD-depressive	289	63	20		
	BD-manic	61	175	9		
	BD-mixed	0	0	0		
	***Xgboost***				**0.799**	**0.795**
	BD-depressive	310	55	16		
	BD-manic	40	183	13		
	BD-mixed	0	0	0		
	***Logistic regression***				**0.771**	**0.756**
	BD-depressive	301	61	16		
	BD-manic	46	174	12		
	BD-mixed	3	3	1		
	***Random forests***				**0.783**	**0.722**
	BD-depressive	301	56	16		
	BD-manic	49	182	13		
	BD-mixed	0	0	0		

Resampling of traindata	Confusion matrix of testdata	BD-Manic (*n* = 350)	BD-depressive (*n* = 238)	BD-Mixed (*n* = 29)	Accuracy	AUC
	***SVM***				**0.737**	**0.735**
	BD-depressive	259	42	18		
	BD-manic	90	196	11		
	BD-mixed	1	0	0		
	***Xgboost***				**0.744**	**0.791**
	BD-depressive	260	38	12		
	BD-manic	82	196	14		
	BD-mixed	8	4	3		
	***Logistic regression***				**0.626**	**0.734**
	BD-depressive	208	35	8		
	BD-manic	65	165	8		
	BD-mixed	77	38	13		
	***Random forests***				**0.747**	**0.762**
	BD-depressive	282	59	18		
	BD-manic	68	179	11		
	BD-mixed	0	0	0		

The bold values presented in Table 3 indicate the machine learning methods and their performance metrics.

**Table 4:** Binary classification performance of four algorithms.

Non-resampling of traindata	Performance of testdata	Binary classification type	AUC	Sensitivity	Specificity
	***SVM***	Manic and depressive	0.874	0.824	0.721
		Manic and mixed	0.662	0.621	0.515
		Mixed and depressive	0.608	0.667	0.514
	***Xgboost***	Manic and depressive	0.885	0.849	0.738
		Manic and mixed	0.736	0.828	0.540
		Mixed and depressive	0.744	0.833	0.534
	***Logistic regression***	Manic and depressive	0.883	0.836	0.729
		Manic and mixed	0.762	0.862	0.544
		Mixed and depressive	0.724	0.733	0.520
	***Random forests***	Manic and depressive	0.866	0.775	0.774
		Manic and mixed	0.740	0.759	0.582
		Mixed and depressive	0.729	0.633	0.666

Resampling of traindata	Performance of testdata	Binary classification type	AUC	Sensitivity	Specificity
	***SVM***	Manic and mixed	0.734	0.759	0.532
		Mixed and depressive	0.734	0.767	0.523
	***Xgboost***	Manic and mixed	0.717	0.690	0.523
		Mixed and depressive	0.732	0.733	0.520
	***Logistic regression***	Manic and mixed	0.755	0.793	0.536
		Mixed and depressive	0.741	0.767	0.533
	***Random forests***	Manic and mixed	0.747	0.759	0.565
		Mixed and depressive	0.750	0.733	0.629

The bold values presented in Table 4 indicate the machine learning methods and their performance metrics.

In the multiclass classification, the best performance in the holdout testing dataset was achieved using the non-resampling training dataset and the Xgboost classifier. This model achieved an overall accuracy of 79.90% and an AUC of 79.5%. The balanced training dataset had the same number of subjects as the original unbalanced dataset, but with a ratio of 1:1:1 for the three clinical states. Although the accuracy of the re-sampling model was slightly reduced, mixed clinical states could be detected more effectively in the LR model (with a 44.8% detecting rate and an AUC of 73.4%).

**Figure 2:**
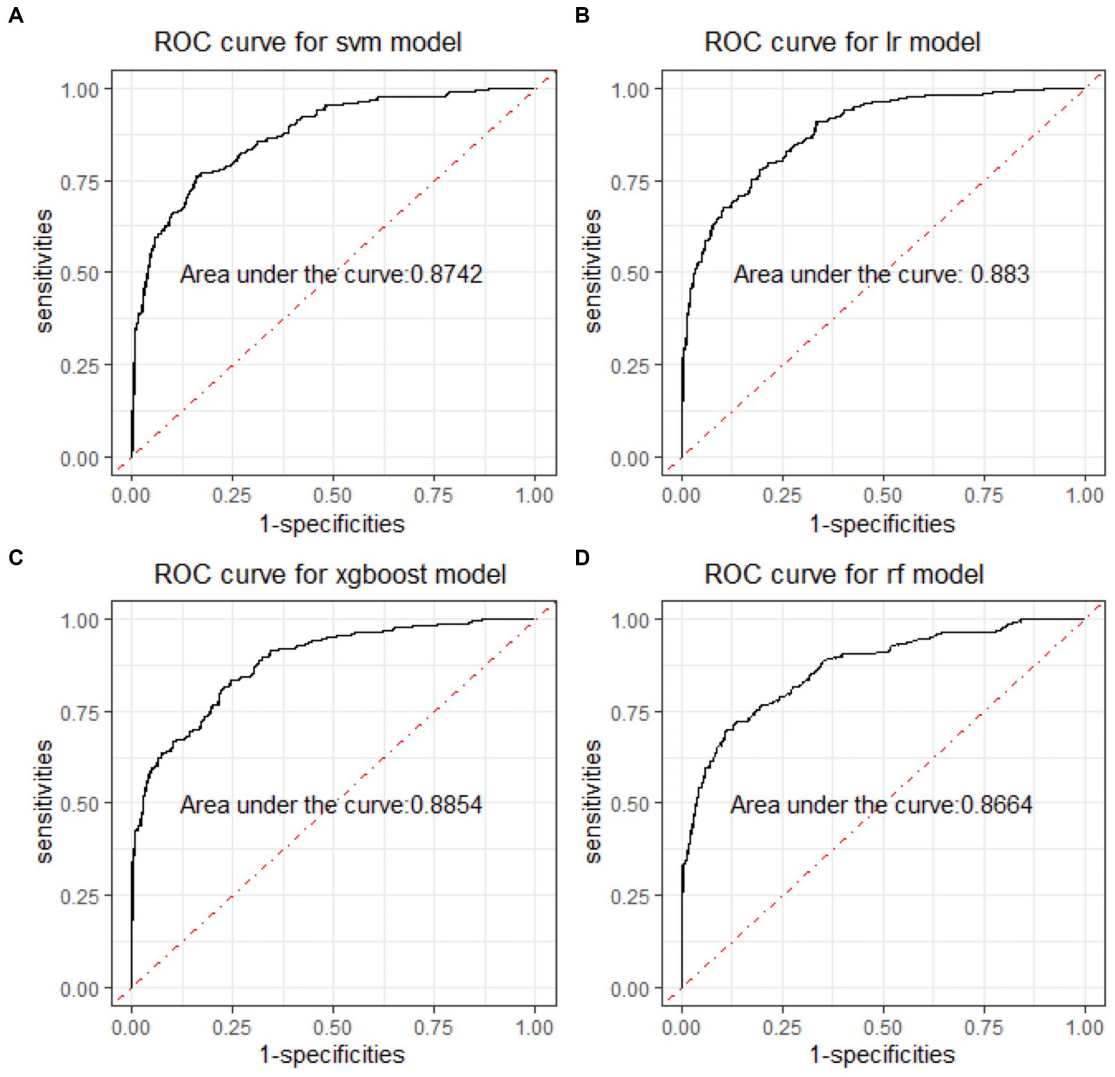
ROC curves of four machine learning models for distinguishing BD manic and depressive clinical states. ROC is the abbreviation of area under the receiver operating characteristics. BD is the abbreviation of bipolar disorder. SVM is the abbreviation of support vector machine. LR is the abbreviation of logistic regression. xgboost is the abbreviation of eXtreme gradient boosting. RF is the abbreviation of random forests.

**Figure 3:**
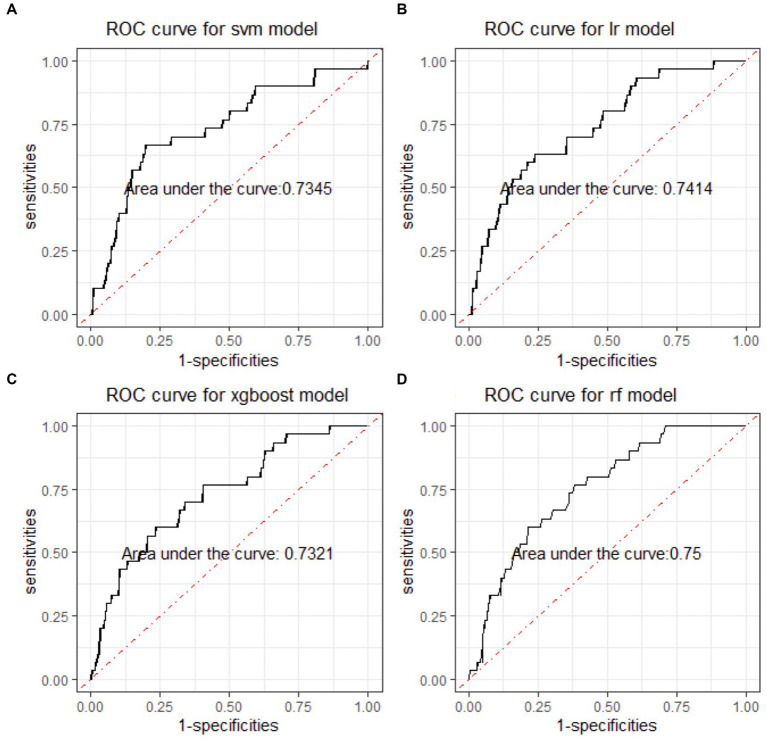
ROC curves of four machine learning models for distinguishing BD mixed and depressive clinical states. ROC is the abbreviation of area under the receiver operating characteristics. BD is the abbreviation of bipolar disorder. SVM is the abbreviation of support vector machine. LR is the abbreviation of logistic regression. xgboost is the abbreviation of eXtreme gradient boosting. RF is the abbreviation of random forests.

**Figure 4:**
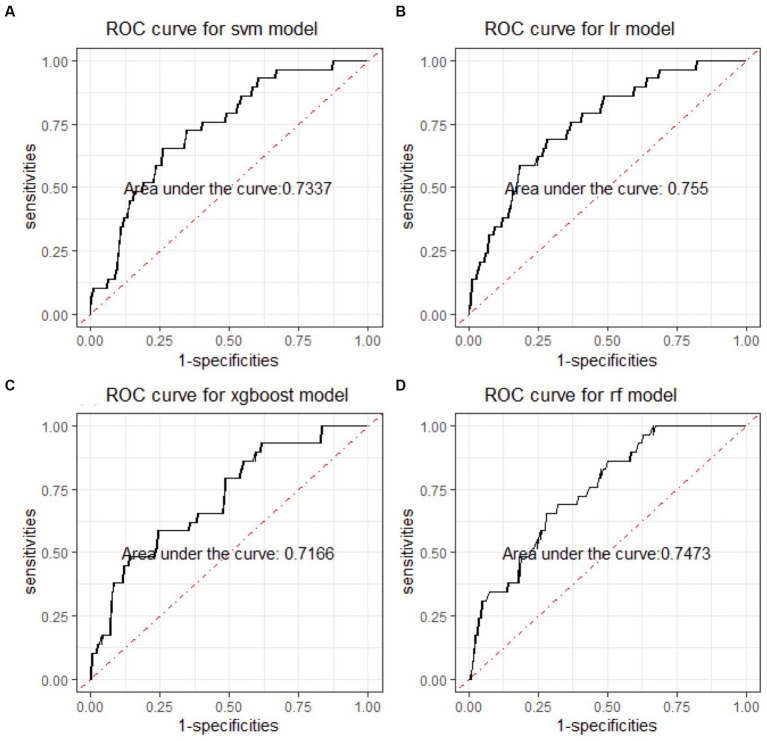
ROC curves of four machine learning models for distinguishing BD manic and mixed clinical states. ROC is the abbreviation of area under the receiver operating characteristics. BD is the abbreviation of bipolar disorder. SVM is the abbreviation of support vector machine. LR is the abbreviation of logistic regression. xgboost is the abbreviation of eXtreme gradient boosting. RF is the abbreviation of random forests.

In the binary classification models, the classifiers performed well for distinguishing between manic and depressive clinical states, with all AUCs exceeding 85% (see Figure 2). The Xgboost classifier emerged as the top performer, achieving an overall AUC of 88.54%, a sensitivity of 84.87% and a specificity of 73.78% in the holdout testing dataset. For distinguishing between BD mixed and depressive clinical states, the Xgboost classifier again emerged as the top performer, achieving an overall AUC of 74.42%, a sensitivity of 83.33% and a specificity of 53.43%. However, in distinguishing between BD manic and mixed clinical states, performance was more variable due to the unbalanced nature of the dataset. The best performance was using the LR classifier, achieving an overall AUC of 76.18%, a sensitivity of 86.21% and a specificity of 54.43%. The classification performance was found to be closely associated with the method of data handling. Similar to the multiclass classification, we also evaluated the performance of re-sampling binary classification (Figures 3, 4). While the performance of Xgboost classifier did not improve using over-sampling, the other three classifiers all showed improvement. Specifically, the SVM classifier’s AUC increased from 66.17% to 73.37% for distinguishing between BD manic and mixed clinical states, and from 60.82% to 73.45% for distinguishing between BD depressive and mixed clinical states.

#### 3.2.2 Important features

**Figure 5:**
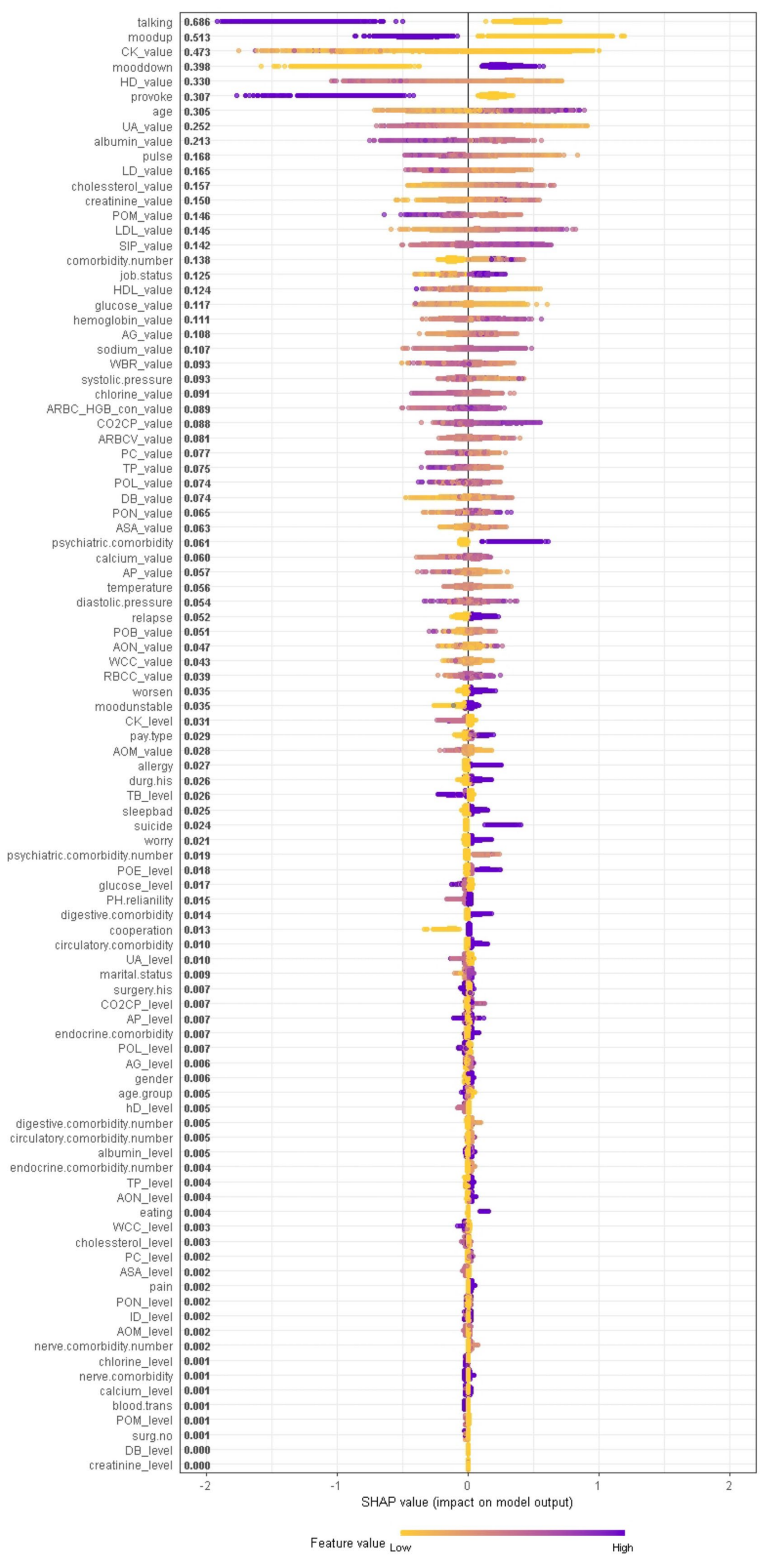
The impact of the input features on predictions of Xgboost in binary classification between BD manic and depressive clinical states.

**Figure 6:**
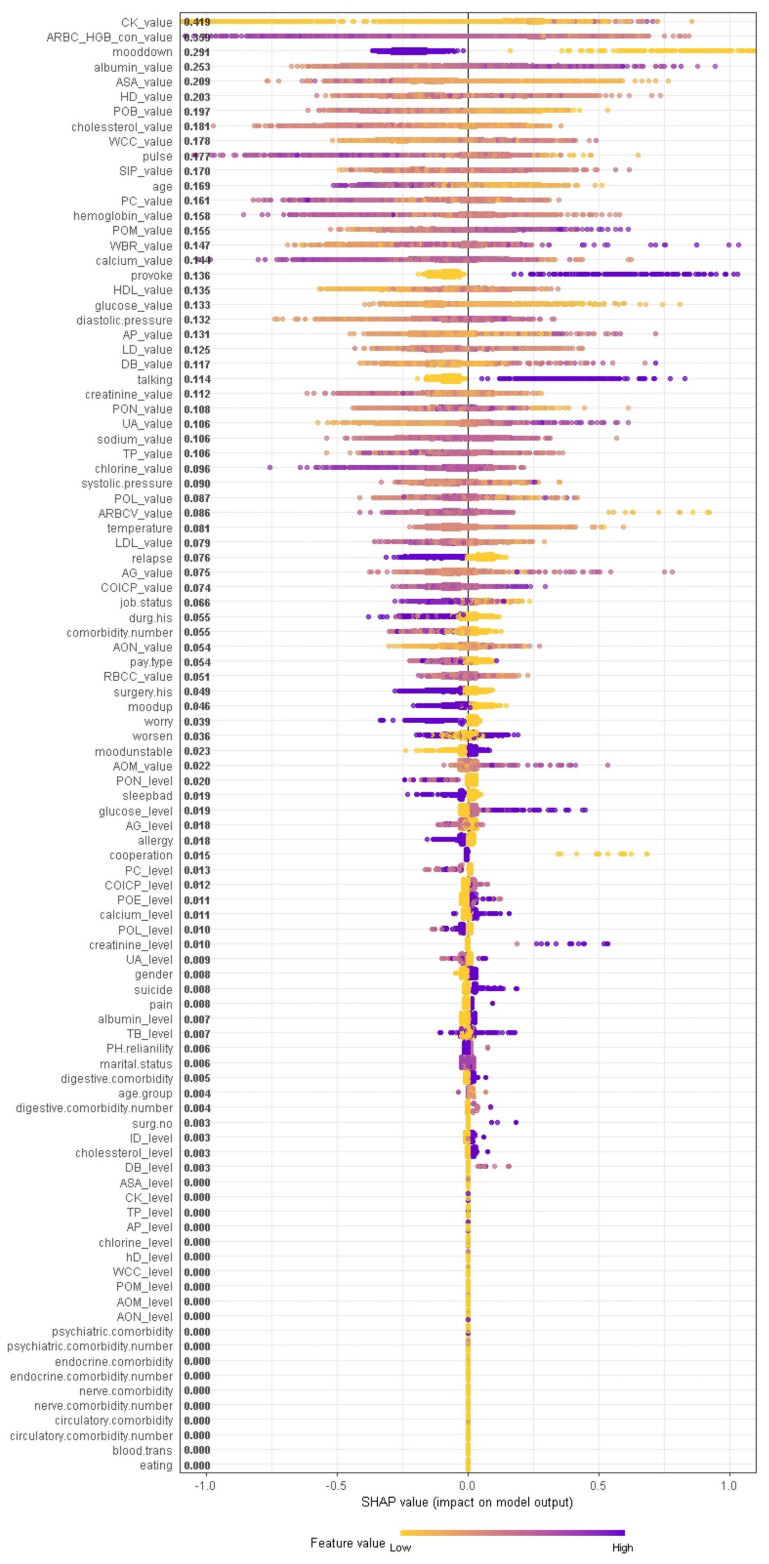
The impact of the input features on predictions of Xgboost in binary classification between BD mixed and depressive clinical states.

**Figure 7:**
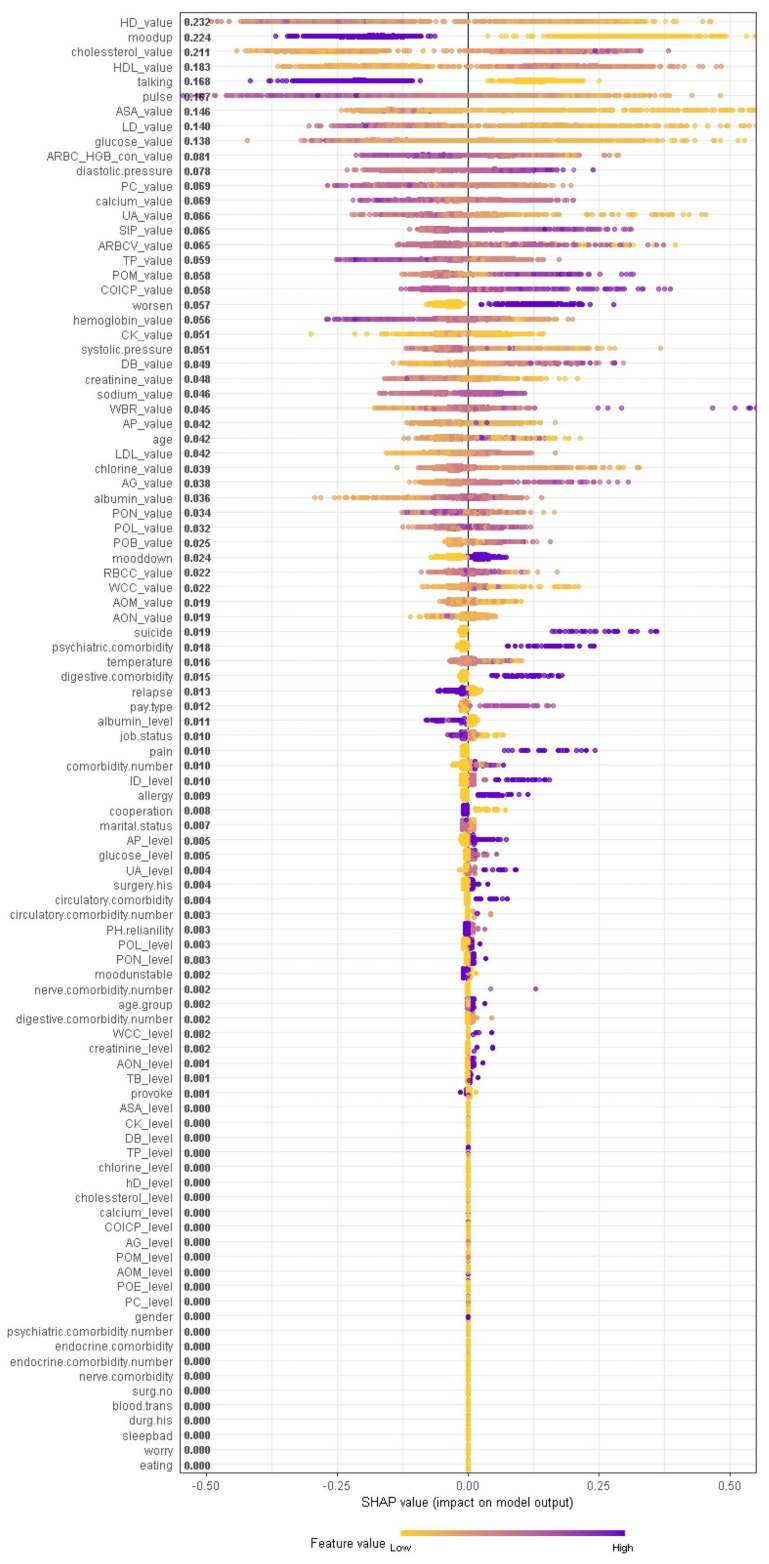
The impact of the input features on predictions of Xgboost in binary classification between BD manic and mixed clinical states.

When considering the contribution of each of the 94 features in the three binary classification models, the feature importance results of three Xgboost models were shown in Figures 5–7. The models included continuous, categorical, and binary features. Continuous and categorical features vary from low to high values, whereas binary features are either present or absent. In Figure 5, each dot represents the impact of a feature on the prediction of BD manic or depressive episodes for one patient in the training set. To be more specific, dots to the right (a SHAP value >0) mean that patients with feature values contributed to a class “1” (BD-depression) decision whereas dots to the left (a SHAP value ≤0) mean that patients with feature values contributed to a class “0” (BD-mania) decision. The color of each dot represents the feature value, with more purple dots indicating higher values and yellower dots indicating lower values. The numerical values next to each feature on the vertical axis represent the mean absolute value of the SHAP values, indicating the relative importance of each feature’s contribution to the predicted value. Larger values indicate a wider distribution of SHAP values. The impact of each feature on the BD depressive or mixed state prediction and the impact of each feature on the BD manic or mixed state prediction were similarly shown in Figures 6, 7 respectively, and dots to the right both mean prediction of BD mixed state. Finally, Supplementary Figures S1–S3 present the relative importance of all features on predictions for the holdout test dataset at the individual patient level.

It should be noted that the presence of talkativeness and elevation of mood drive predictions towards a manic state, while the absence of these symptoms influences predictions towards a depressive state. This is consistent with traditional clinical diagnostic criteria, which consider elevation of mood and talkativeness as key symptoms distinguishing mania from depression. In addition to symptom markers, the age at admission significantly contributed to predictions, with older age predicting BD-depression and younger age predicting BD-mania, reflecting the age distribution of BD episodes. Notably, comorbidity conditions and job status also had a significant impact. Biological laboratory test indicators and vital signs were also identified as important features, ranking among the top features overall, including myocardial enzyme markers (creatine kinase, hydroxybutyrate dehydrogenase, lactate dehydrogenase), non-enzymatic antioxidant uric acid, liver biochemistry markers (albumin), vital signs (pulse), serum metabolism markers (cholesterol, creatinine, low/high density lipoprotein, glucose), markers of inflammation (percentage of monocyte), serum inorganic phosphorous, and red blood cell markers (hemoglobin), etc., suggesting the overall health of other tissues or organs is closely corresponds to the pathophysiological mechanisms of BD.

In contrast, symptom markers are not able to predominantly contribute to the classification of BD mixed and depressive (manic) clinical states due to overlapping neuropsychological symptomatology; instead, biological markers contribute the most in such scenarios. Particularly, in the classification of BD mixed and depressive episodes (Figure 6), creatine kinase, average red blood cell HGB concentration, albumin and aspartate aminotransferase were identified as the most significant biological markers. Additionally, the only symptom marker among the top 5 contributing features was a lowering of mood (depression-related symptoms are less typical in mixed episode than in depressive episode). In the classification of BD mixed and manic clinical states (Figure 7), hydroxybutyrate dehydrogenase, cholesterol and high density lipoprotein were identified as the most contributed biological markers. Furthermore, elevation of mood and talkativeness were two symptom markers among the 5 top contributing features (mania-related symptoms are less typical in mixed episode than in manic episode).

#### 3.2.3 Feature interactions

**Figure 8:**
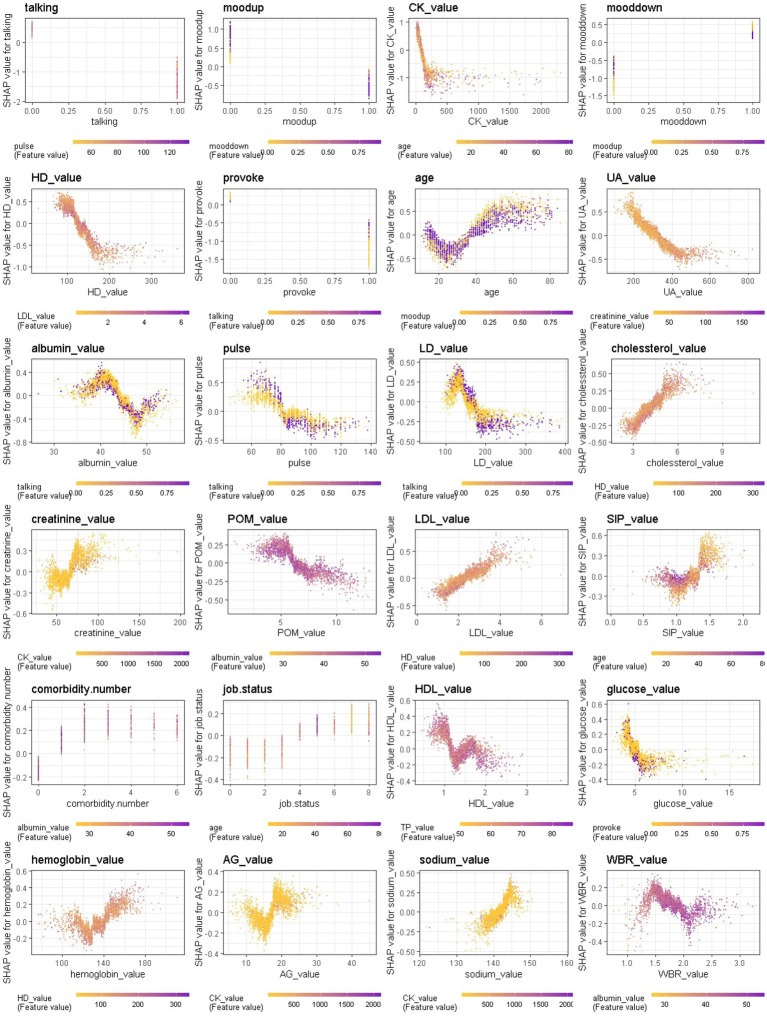
Interaction effects of 24 top important features of Xgboost in binary classification between BD manic and depressive clinical states.

**Figure 9:**
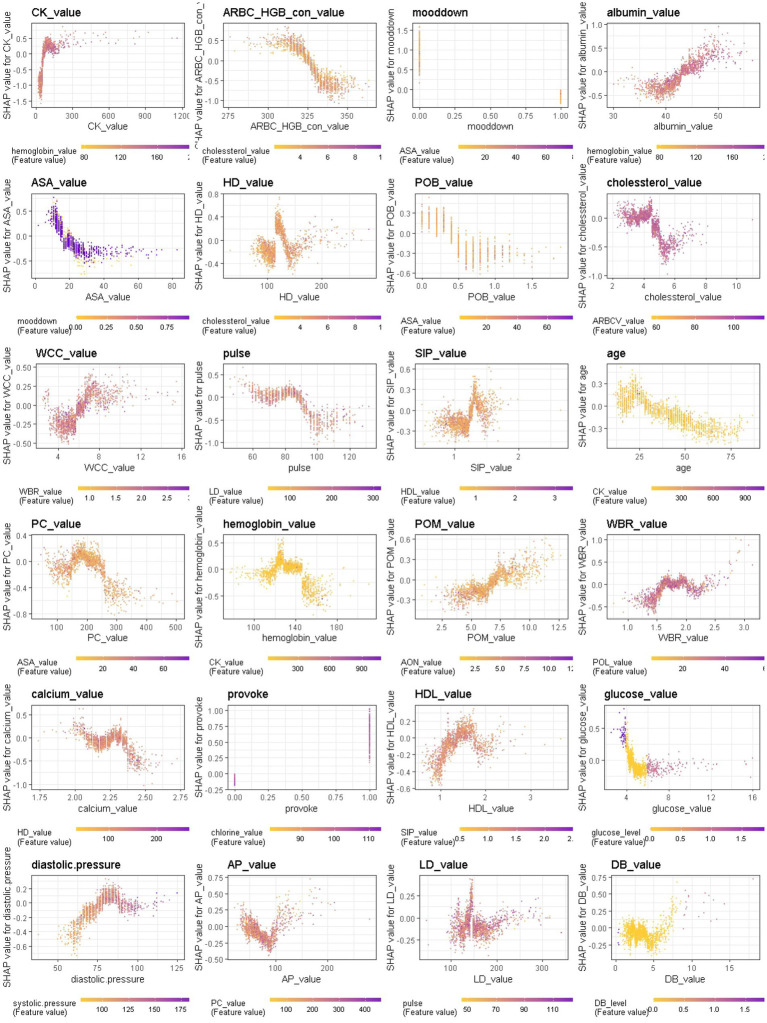
Interaction effects of some important features of Xgboost in binary classification between BD mixed and depressive clinical states.

**Figure 10:**
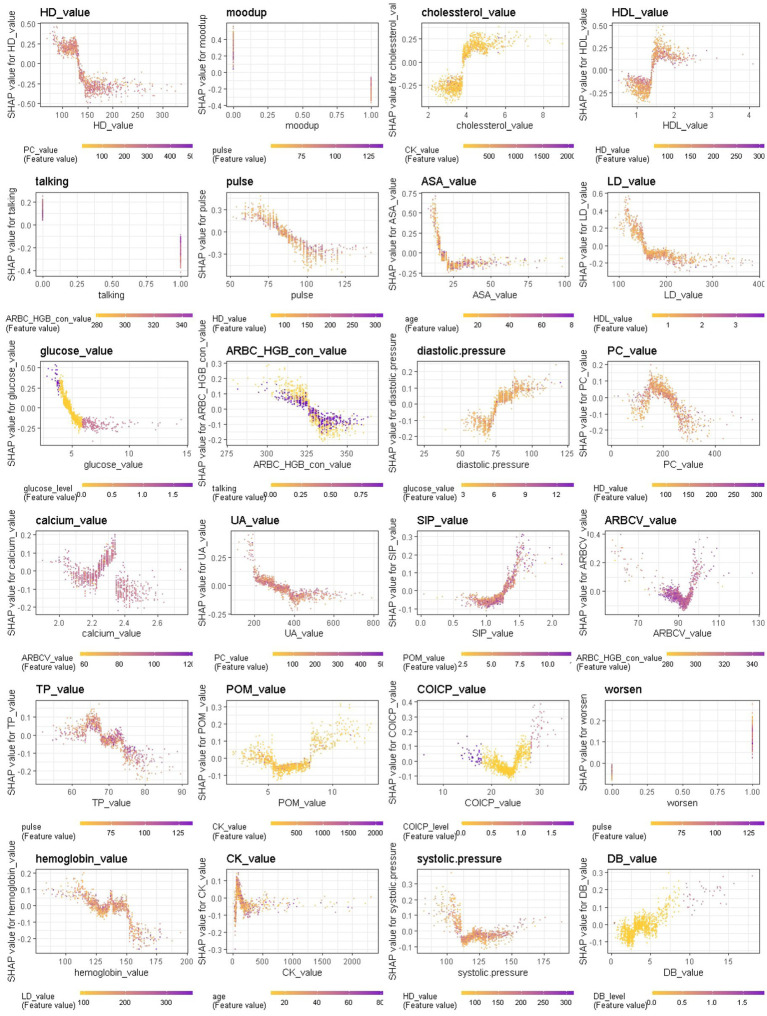
Interaction effects of some important features of Xgboost in binary classification between BD mixed and manic clinical states.

We further provide a visual example of how the top 24 features in each model interact (Figures 8–10). The figures also show the varying trend of the SHAP value of each feature. In each figure, the *X*-axis represents the feature values, and the *Y*-axis represents the SHAP value of the specific feature on the *X*-axis. The color represents the value of the feature that interacts with the specific feature on the *X*-axis; the more purple the dot, the higher the interacted feature value, while the yellower the dot, the lower the interacted feature value. In Figure 8, when considering the continuous features like creatine kinase, hydroxybutyrate dehydrogenase, and uric acid, the SHAP value (relative risk of BD-depression prediction) first decreases with increasing values of these three features and then stabilizes gradually. Additionally, age, albumin, pulse, and lactate dehydrogenase exhibit clear threshold effects. For patients under 35 years old, BD-mania prediction is the main risk, which first increases with age and then decreases regardless of whether there is an elevation of mood symptom. For patients over 35 years old, BD-depression prediction is the main risk, and absence of the elevation of mood symptom has a higher relative risk than patients with such symptoms. The risk increases smoothly with age regardless of whether there is the elevation of mood symptom.

### 3.3 Additional analyses to assess the effect of confounders

Since this was a retrospective study, additional analyses included assessing the longitudinal evolution of patients with different BD clinical states: (1) investigating whether there are significant differences in the physiological and biological markers at the onset of different BD clinical states within an individual longitudinally; (2) validating whether ML models based on EMR data could be effective for predicting the longitudinal evolution of patients’ clinical states. Supplementary Figure S4 showed the flow of longitudinal cohort construction. For the 3,085 BD records, 369 of them had more than once admission records for BD at WCH. Among them, 203 were first diagnosed with BD depressive episodes, 153 were first diagnosed with BD manic episodes, and 13 were first diagnosed with BD mixed episodes. Through longitudinal observation of these patients, we identified the evolution of each patient’s subsequent clinical states (see Supplementary Figure S4).

Based on the three cohorts, follow-up analyses were conducted to investigate whether there were significant differences in the physiological and biological markers of 35 patients first diagnosed with BD depressive episodes and subsequently diagnosed with BD manic episodes. Thre results showed that some laboratory biological markers did present significant differences within an individual longitudinally in different BD clinical states (for details, refer to Supplementary Information 1.2). As shown in Supplementary Table S2, alanine aminotransferase, creatine kinase, lactate dehydrogenase, uric acid, hydroxybutyrate dehydrogenase, white blood cell count (leukocyte count), absolute value of monocyte, etc. were significant with *p*-value less than 0.05. The finding suggested that laboratory biological markers can indeed reflect the biological differences of different BD clinical states, while controlling the impact of individual confounders as much as possible.

Moreover, additional validation in ML models for predicting the longitudinal evolution of patients’ clinical states was conducted. The results showed a test AUC of 0.559 for predicting the longitudinal conversion from BD depressive episodes to BD manic episodes, and a test AUC of 0.807 for predicting the longitudinal conversion from BD manic episodes to BD depressive episodes (Supplementary Information 1.2; Supplementary Figure S4). These findings suggested that neuropsychological symptomatology, comorbidities, vital signs, and blood laboratory measures could predict different BD clinical states in both cross-sectional and longitudinal study settings. However, additional key information should be included into the models to enhance the prediction of conversion from BD depressive episodes to BD manic episodes. Due to the small number of patients in BD mixed clinical state, we did not include an analysis of longitudinal evolution for this subset of patients.

## 4 Discussion

The present study investigated the possibility of distinguishing between BD manic, depressive, and mixed clinical states using multiple ML classifiers based on structured and unstructured EMR data. All patients were interviewed and diagnosed by a team of psychiatrists and psychologists in the studied hospital. We designed four machine learning algorithms (LR, SVM, RF, Xgboost) and evaluated their performance in non-resampling and resampling multiclass/binary classifications due to the imbalance nature of different clinical states (especially the mixed state).

In non-resampling multiclass classification, the Xgboost classifier emerged as the best performer, achieving an overall accuracy of 79.90% and an AUC of 79.5% in the holdout testing dataset. However, it correctly identified only one of 29 subjects diagnosed with mixed state. In the resampling setting, the LR model emerged as the most effective classifier for detecting mixed states, with a detection rate of 44.8%. When considering binary classification models for distinguishing between manic and depressive states, most classifiers performed well, with all AUCs exceeding 85%. The Xgboost model performed the best achieving an overall AUC of 88.54%. In non-resampling scenarios, Xgboost also excelled at distinguishing between mixed and depressive states, with an AUC of 77.42%, while LR emerged as the top performer for distinguishing between mixed and manic states, with an AUC of 76.18%. These classification models were trained using information that can be easily collected from patients prior to hospitalization. With sufficient data on a reasonable number of patients, these algorithms could serve as an additional tool in mental health services to direct BD diagnosis and predict the course of illness in a data-driven manner. However, it is important to note that these models were not designed to replace proper clinical evaluation. To the best of our knowledge, this is the first comprehensive dissection of clinical and biological heterogeneity in BD clinical states, considering overlapping neuropsychological symptomatology, vital signs, comorbidity, and blood laboratory indicators. We used these features to predict BD clinical states, and our results were highly comparable with previous studies. Overall, we found that the different BD clinical states have distinct profiles of specific episode-related effects.

In this study, it was found that BD is more prevalent in women than in men, especially the BD mixed state. Among individuals diagnosed with BD mania, a gender difference was observed with more males than females in this state, whereas more females than males were found among individuals diagnosed with BD depression or mixed state. Men tend to exhibit hyperactivity, grandiosity, and engage in risky behavior, while women tend to report more racing thoughts and distractibility (18). Levels of mood-related depression symptoms (i.e., lowering of mood, mood instability) were similar in depressed and mixed states, and both were significantly more depressed than manic state. Moreover, manic, and mixed states differed in terms of mania severity, as demonstrated by mood-related mania symptoms (i.e., elevation of mood, talkativeness, and irritability), and both were significantly more manic than the depressed state. The finding is also reported by Singh et al. (21), in a small sample cross-sectional study. Anxiety-related symptom (i.e., worry) and sleep-related symptom (i.e., poor sleep) were similar in mixed and manic states, and symptoms of anxiety and bad sleep in both states were significantly less common as those in depressed state. Singh et al. (21) reported that anxiety symptoms were particularly severe in mixed episodes. Differences in suicidality and psychomotor abnormalities (i.e., appetite disturbances) were observed with the mixed state showing more severe symptoms than depressed and manic states, while depressed subjects presented with more somatic symptoms (i.e., pain) than those with the mixed and manic states. No statistically significant differences in psychotic symptoms were found among the three BD clinical states. Reported rates of comorbidity ranged from 0.4% to 14.7%. The depressed state presented with more comorbid conditions than the manic and mixed states, including the psychiatric, endocrine, digestive, and circulatory systems. The number of psychiatric comorbidities was similar in depressed and mixed states, and both states have significantly more psychiatric comorbid conditions than manic state.

In current clinical practice settings, patients’ mood is commonly assessed through clinician-administered rating scales and questionnaires, physiological and biological parameters are not used for this purpose. However, recent findings suggest that these parameters may be sensitive to clinical states and could serve as predictors of mood state changes. Particularly, higher levels of body temperature, pulse, and systolic and diastolic blood pressures were observed during mania in BD, which may be associated with increased energy, lability, and irritability. When comparing the patterns with respect to the BD mania/depression classification and the BD mania/mixed state classification, we found that talkativeness, elevation of mood, hydroxybutyrate dehydrogenase, uric acid, pulse, lactate dehydrogenase, cholesterol and glucose contributed most in both models. In BD manic episodes, mean value of cholesterol was lower than in other two states, while mean values of the remaining markers were higher than in other two states, indicating that clinical and biological heterogeneity in BD manic episodes can be further understood through these markers. This finding, that uric acid levels are higher in BD manic episodes compared to BD depressive episodes, has also been reported in reference (23).

**Figure 11:**
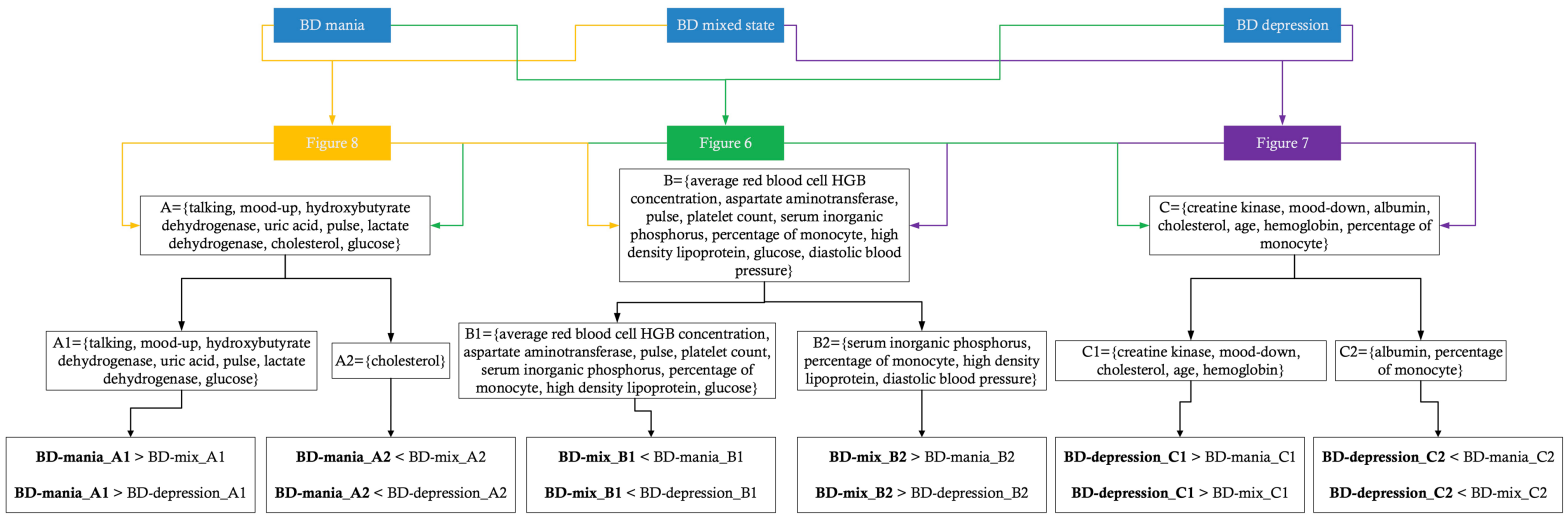
Potential markers for each clinical state of BD.

When comparing the patterns with respect to the BD depression/mixed state classification and the BD mania/mixed state classification, we found that average red blood cell HGB concentration, aspartate aminotransferase, pulse, platelet count, serum inorganic phosphorus, percentage of monocyte, high density lipoprotein, glucose and diastolic blood pressure contributed most to both models. In BD mixed episodes, the mean values of average red blood cell HGB concentration, aspartate aminotransferase, pulse, platelet count, and glucose were lower than in other two states, while mean values of serum inorganic phosphorus, percentage of monocyte, high density lipoprotein, and diastolic blood pressure were higher than in other two states. By monitoring these markers, physicians can better identify patients in BD mixed episodes. When comparing the patterns with respect to the BD depression/mixed state classification and the BD mania/depression classification, we found that creatine kinase, lowering of mood, albumin, cholesterol, age, hemoglobin, percentage of monocyte contributed most in both models. In BD depressive episodes, mean values of creatine kinase, lowering of mood, cholesterol, age, and hemoglobin were higher than in other two states, while mean values of albumin and percentage of monocyte were lower than in other two states (see Figure 11). These results suggest that physiological and biological parameters may serve as potential biomarkers for differentiating BD manic, depressive, and mixed clinical states. To the best of our knowledge, this is the first systematic evidence of these patterns of difference which clinicians and investigators alike may be able to utilize to aid both in better diagnosing BD mood states and, by extension, illness course prediction and establishing and implementing treatment plans.

Notably, the myocardial enzyme spectrum is a biomarker for diagnosing cardiovascular diseases and also contributed most to our classification models. Elevated concentrations of myocardial enzymes in both BD and cardiovascular diseases may play an important role in identifying the etiology and pathogenesis of BD. Evidence suggests that BD is associated with a higher risk of the co-occurrence with cardiovascular diseases, which could be due to genetic (37) or biological alterations, including immune-inflammatory pathways and oxidative stress pathways that are closely related (38). It is acknowledged that excessive oxidative stress among BD patients exists (22), with an imbalance between oxidant and antioxidant species (such as the antioxidant enzymes catalase and the non-enzymatic antioxidant uric acid, bilirubin). Our results also suggest that uric acid and direct bilirubin contributed significantly to distinguishing between BD clinical states both in the cross-sectional and longitudinal study settings. Although many works point to alterations in antioxidant enzymes among patients with BD, however, no study reported the relationship between the myocardial enzymes and BD episodes according to the polarity of individuals with BD. Because there is no single, reliable biomarker, future prospective studies to validate a novel possibility for myocardial enzymes being used for diagnosis of BD are needed.

Additionally, we found that features interact in complex ways, and the ambiguity of these features further emphasizes the need for machine learning-based diagnostic decision support. Although the models evaluate the contributions of individual features differently, it is a combination of several features that provides specific classification performance. We observed that the features interact in a complex non-linear manner, such as the compensated interaction effect between elevation of mood and lowering of mood symptoms, and the threshold effect between continuous and binary variables (i.e., age with elevation of mood, albumin with talkativeness, pulse with talkativeness and lactate dehydrogenase with talkativeness) when distinguishing between manic and depressive states. The most important features varied across different classification scenarios (manic/mixed states, depressive/mixed states, and manic/depressive states), and were consistent among different models trained in the same scenario. This reinforces the application of ML models to reveal hidden layers of information in clinical data collected from patients.

## 5 Limitations

There are several limitations of this study. Firstly, the entire study was conducted retrospectively using a single and imbalanced sample collected from a large hospital’s EMR data. There is a high probability of selection bias, which could hamper the generalizability of the ML algorithms trained in this dataset, despite all appropriate procedures being followed to prevent overfitting. External validation based on a larger sample size and multi-site samples could have reduced the uncertainty of some of our models, particularly regarding the performance of models resampling the positive and negative classes. Secondary, it should be emphasized that discharge diagnoses were used as the outcome labels of our classification models, and patients’ information collected prior to admission formed the basis of the predictors for the classification models. In this research setting, the utility and representativeness are diminished by limiting the sample to admitted patients, so we are only considering severe bipolar disorder. Although the chief complaint data was analyzed by NLP technology to extract patients’ symptoms and current mood states, clinical severity of episodes/symptoms was not considered due to the limitations of the EMR data itself. Thirdly, current nosology systems in psychiatry have limitations and were not designed to predict risk of future mood episodes. New systems might help overcome this challenge by refining current concepts for psychiatric disease classification and identifying potential diagnostic biomarkers. Additionally, it has been reported that mood stabilizers, such as lithium and valproate, possess antioxidant and anti-inflammatory properties in bipolar patients. The use of these drugs as a treatment for patients could serve as a reliable predictor of bipolar disorder, leading one to expect the identification of elevated glucose concentrations. Given that one of the purposes of this study is to develop objective evaluation measures to establish a data-driven diagnostic decision support model, only the information available for admission diagnosis were considered. Nevertheless, whether patients are undergoing psychotropic drug treatment may influence the independent contribution of biological laboratory indicators such as uric acid, glucose, and so on, to the performance of ML models. It is also challenging to exclude the possible effect of other unknown factors on levels of serum laboratory indicators. Clinically, we believe that the limitations suggest that our model approach would be insufficient for directly using as a clinical diagnosis tool without further investigation. However, we believe that because our model was constructed directly from EMR data, integrating it into an EMR-based systemwide clinical decision support program would be more practical than if the model were created using data that needed to be collected outside the EMR.

## 6 Conclusion

Using a longitudinal design that incorporated within-subject comparisons between clinical states, we investigated whether neuropsychological symptomatology, comorbidity, vital signs, and blood laboratory indicators can predict distinct BD states, specifically BD-mania, BD-depression, and BD-mixed state. We employed explainable machine learning techniques to analyze the data. Our findings contribute to a better understanding of the clinical, physiological, and biological heterogeneity among BD clinical states. We found evidence that specific combinations of features could serve as potential diagnostic markers for each clinical state of BD. Finally, larger studies are needed to map biological risk factors more precisely to clinical states. The identification of underlying heterogeneity across distinct BD clinical states is a well-recognized challenge. Nevertheless, such efforts are critical in helping classify psychiatric disorders more accurately and may contribute to psychiatric clinical classification systems with a more biologically informed nosological system.

## Data availability statement

The data used to support the findings of this manuscript are restricted by the West China Hospital, to protect patient privacy and avoid legal and ethical risks. Data are available from West China Hospital for researchers who meet the criteria for access to confidential data.

## Ethics statement

The Ethics Committee of the West China Hospital, Sichuan University approved the research. The data are anonymous, and the requirement for informed consent was waived by Institute Review Board (IRB) of West China Hospital, Sichuan University. The protection and treatment of patient data in our research comply with the Helsinki Declaration.

## Author contributions

TZ and WZ designed the study and wrote the protocol. TZ, CY, and YH established the database for this study. TZ and RK analyzed the data. TZ wrote the manuscript. MY, LL, and WZ provided critical review and revisions of the manuscript. All authors contributed to the article and approved the submitted version.
