# Supplementary material for: Dissecting clinical and biological heterogeneity in clinical states of bipolar disorder: a 10-year retrospective study from China
Source: Front Psychiatry. 2023 Dec 21;14:1128862. doi: 10.3389/fpsyt.2023.1128862 (PMC10764613; doi:10.3389/fpsyt.2023.1128862)
Supplement: Supplementary file 1 [file Data_Sheet_1.docx]

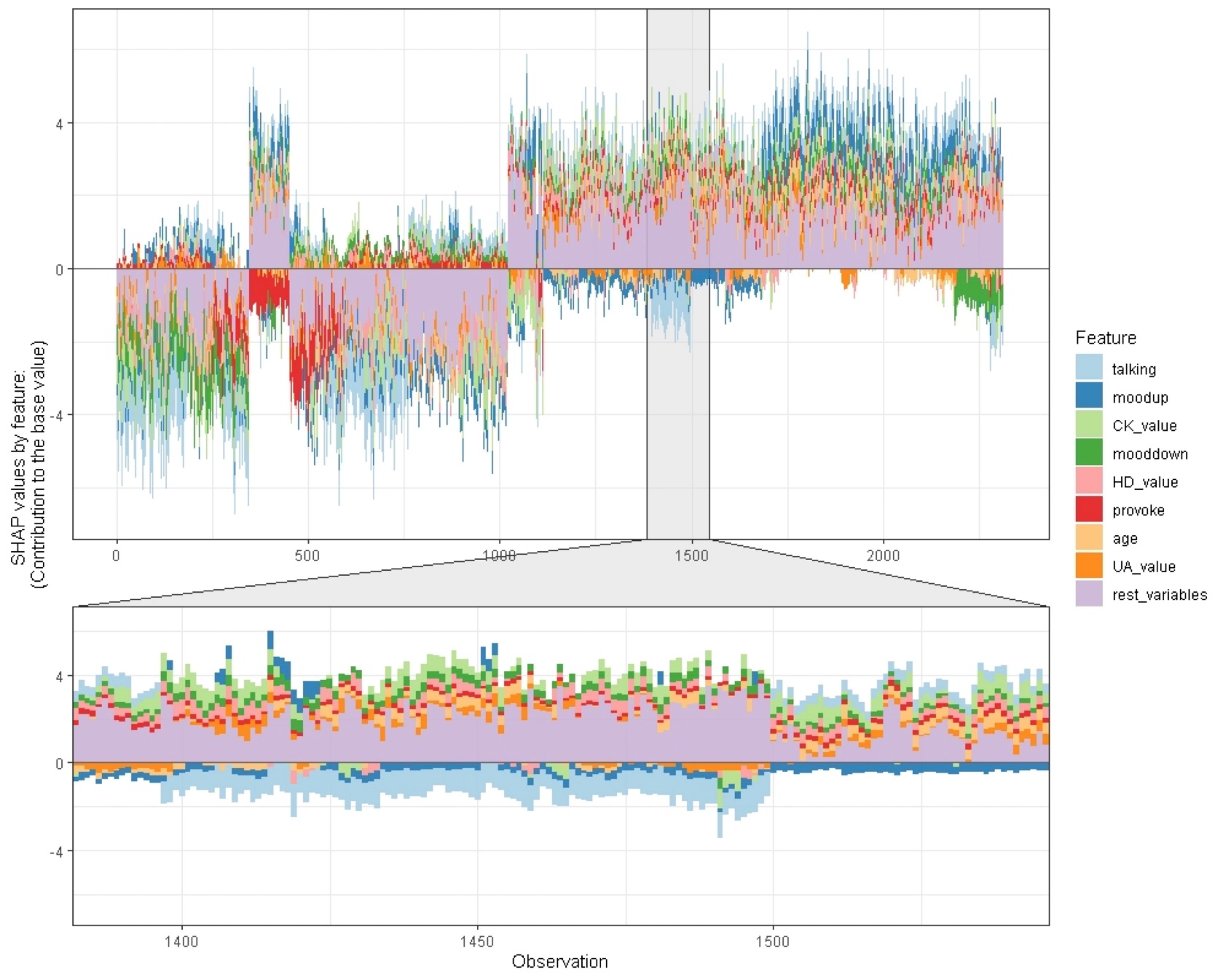


Figure S1: Individual predictions of Xgboost in binary classification between BD manic and depressive clinical states.


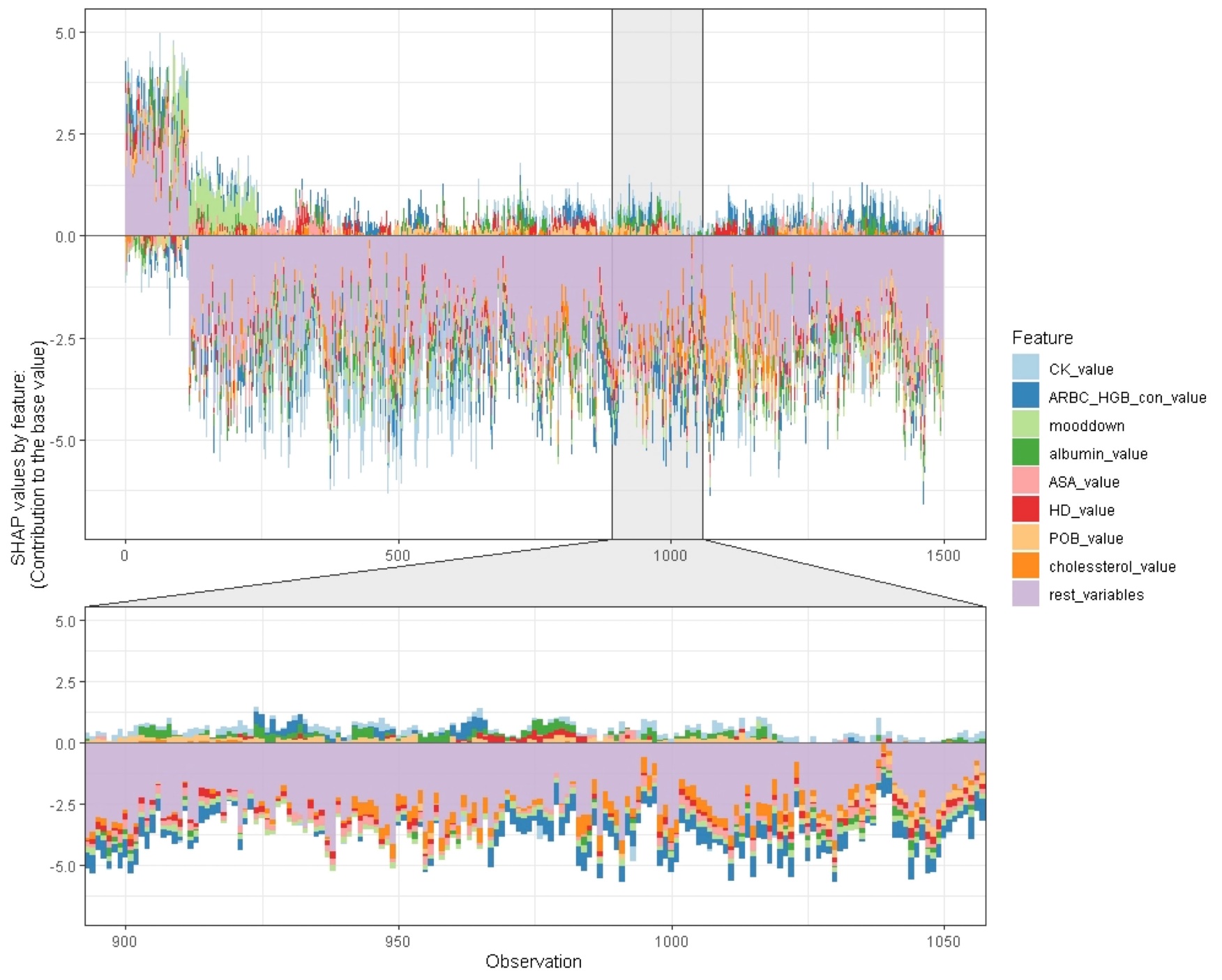


Figure S2: Individual predictions of Xgboost in binary classification between BD mixed and depressive clinical states.


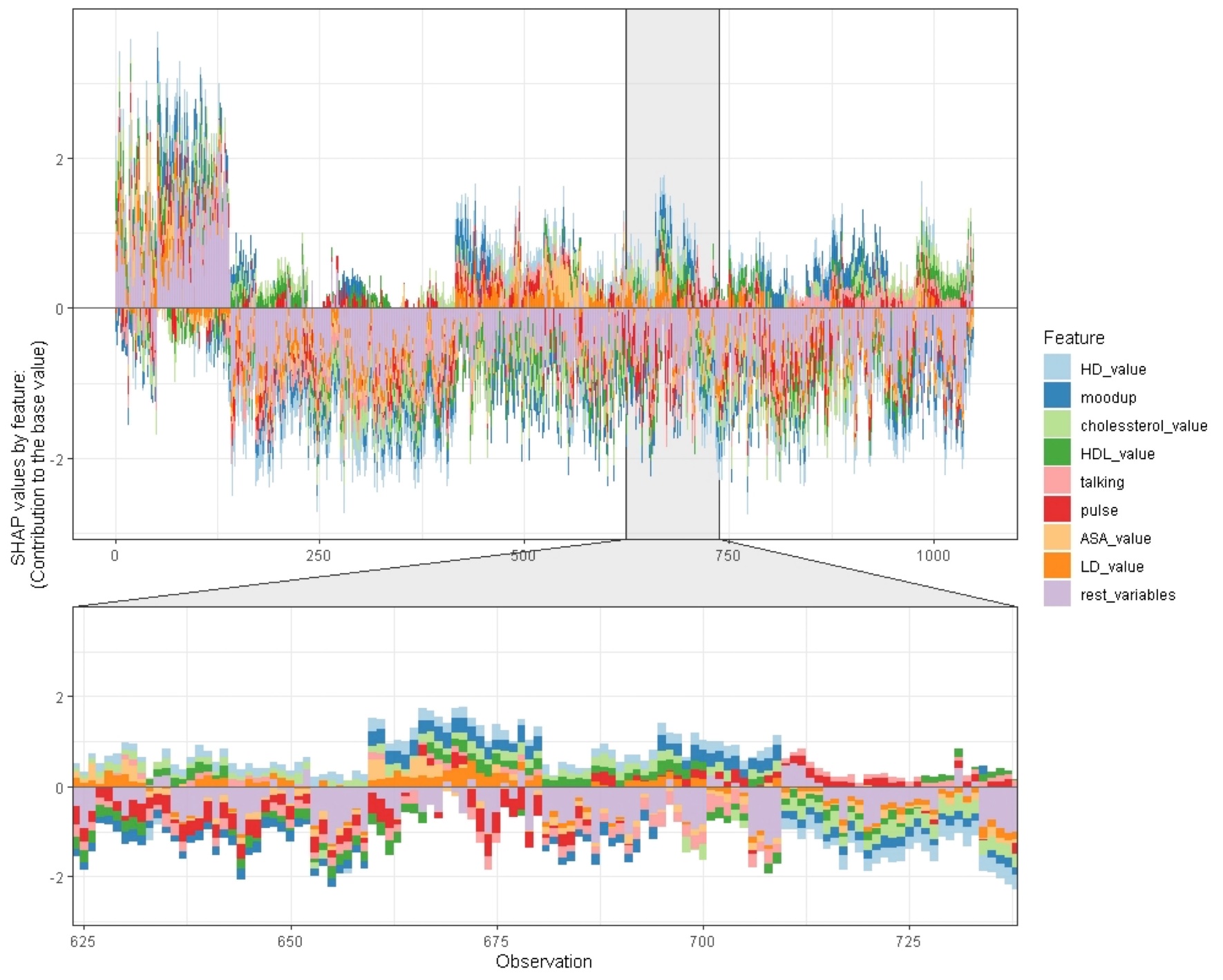


Figure S3: Individual predictions of Xgboost in binary classification between BD manic and mixed clinical states.
