# Supplementary material for: Dissecting clinical and biological heterogeneity in clinical states of bipolar disorder: a 10-year retrospective study from China
Source: Front Psychiatry. 2023 Dec 21;14:1128862. doi: 10.3389/fpsyt.2023.1128862 (PMC10764613; doi:10.3389/fpsyt.2023.1128862)
Supplement: Supplementary file 2 [file Data_Sheet_2.docx]

**Supplementary Information**

- 1. **Features**

In this study, the database consists of a representative set of items to record all information generated during a patient’s hospitalization. Various categories of features were extracted from the original medical records, such as sociodemographic information, basic information about hospitalization, vital signs based on basic body check at admission, information about past illness history and life behavior, information about treatment patterns, and text data (chief complaint, discharge summary and medical record summary). The details of recruited features were concluded in the following Table S1.

Table S1 Features recruited into the raw data of this study

| **Variable name** | **Variable interpretation** | **Data type** |
| --- | --- | --- |
| PADMNO | Patient admission number (unique for each admission record of each patient) | factor |
| gender | Gender | factor |
| age.group | Age group | factor |
| marital.status | Marital status | factor |
| job.status | Job status | factor |
| ethnicity | Nationality | factor |
| out_diag_code | Principal diagnosis code | factor |
| out_diag_name | Principal diagnosis name | factor |
| pay.type | Type of payment | factor |
| pat.source | Source of patient |  |
| chief_complaint | Chief complaint | factor |
| mooddown | Whether the patient has a symptom of lowering of mood | factor |
| moodup | Whether the patient has a symptom of elevation of mood | factor |
| moodunstable | Whether the patient has a symptom of mood instability | factor |
| talking | Whether the patient has a symptom of talkativeness | factor |
| sleepbad | Whether the patient has a symptom of bad sleep | factor |
| pain | Whether the patient has a symptom of painful | factor |
| worry | Whether the patient has a symptom of worry | factor |
| eating | Whether the patient has a symptom of appetite disturbances | factor |
| hearing | Whether the patient has a symptom of auditory hallucination | factor |
| suicide | Whether the patient has a symptom of suicide ideation | factor |
| provoke | Whether the patient has a symptom of provoke | factor |
| relapse | Whether the patient has a recurrence of above symptoms | factor |
| worsen | Whether the patient has a worsen of above symptoms | factor |
| temperature | Physical examination: to record a patient's body temperature | numeric |
| pulse | Physical examination: to record a patient's pulse | numeric |
| diastolic.pressure | Physical examination: to record a patient's diastolic blood pressure (DBP) | numeric |
| systolic.pressure | Physical examination: to record a patient's systolic blood pressure (SBP) | numeric |
| breathing | Physical examination: to record a patient's breathe | numeric |
| nutrition | Physical examination: to record a patient's nutrition status | factor |
| cooperation | Physical examination: to record a patient's cooperation status | factor |
| allergy | Past illness history: History of allergy | factor |
| blood.trans | Past illness history: History of blood transfusion | factor |
| drug.his | Past illness history: History of drug use | factor |
| surgery.his | Past illness history: History of surgery | factor |
| comorbidity.number | Number of comorbidities | factor |
| respiratory.comorbidity.number | Number of respiratory comorbidity | factor |
| circulatory.comorbidity.number | Number of circulatory comorbidity | factor |
| digestive.comorbidity.number | Number of digestive comorbidity | factor |
| nervous.comorbidity.number | Number of nervous comorbidity | factor |
| endocrine.comorbidity.number | Number of endocrine comorbidity | factor |
| psychiatric.comorbidity.number | Number of psychiatric comorbidity | factor |
| respiratory.comorbidity.number | Number of respiratory comorbidity | factor |
| psychiatric.comorbidity | Whether the patient has a comorbidity of psychiatric diseases | factor |
| endocrine.comorbidity | Whether the patient has a comorbidity of endocrine diseases | factor |
| nervous.comorbidity | Whether the patient has a comorbidity of nervous diseases | factor |
| digestive.comorbidity | Whether the patient has a comorbidity of digestive diseases | factor |
| circulatory.comorbidity | Whether the patient has a comorbidity of circulatory diseases | factor |
| respiratory.comorbidity | Whether the patient has a comorbidity of respiratory diseases | factor |
| cancer.comorbidity | Whether the patient has a comorbidity of cancer | factor |
| RBC_CV_value | Routine blood examination: Red blood cell volume distribution width CV | numeric |
| RBC_CV_level | Routine blood examination: Level of red blood cell volume distribution width CV | factor |
| RBC_SD_value | Routine blood examination: Red blood cell volume distribution width SD | numeric |
| RBC_SD_level | Routine blood examination: Level of red blood cell volume distribution width SD | factor |
| WBCC_value | Routine blood examination: White blood cell count (Leukocyte count) | numeric |
| WBCC_level | Routine blood examination: Level of white blood cell count (Leukocyte count) | factor |
| POM_value | Routine blood examination: Percentage value of monocyte | numeric |
| POM_level | Routine blood examination: Level of percentage value of monocyte | factor |
| AOM_value | Routine blood examination: Absolute value of monocyte | numeric |
| AOM_level | Routine blood examination: Level of absolute value of monocyte | factor |
| RBCC_value | Routine blood examination: Red blood cell count (Erythrocyte count) | numeric |
| RBCC_level | Routine blood examination: Level of red blood cell count (Erythrocyte Count) | factor |
| hematocrit_value | Routine blood examination: Hematocrit value | numeric |
| hematocrit_level | Routine blood examination: Level of hematocrit value | factor |
| POL_value | Routine blood examination: Percentage value of lymphocyte | numeric |
| POL_level | Routine blood examination: Level of percentage value of lymphocyte | factor |
| AOL_value | Routine blood examination: Absolute value of lymphocyte | numeric |
| AOL_level | Routine blood examination: Level of absolute value of lymphocyte | factor |
| ARBC_HGB_value | Routine blood examination: Average value of red blood cell (erythrocyte) HGB | numeric |
| ARBC_ HGB_level | Routine blood examination: Level of average value of red blood cell (erythrocyte) HGB | factor |
| ARBC_HGB_con_value | Routine blood examination: Average value of red blood cell (erythrocyte) HGB concentration | numeric |
| ARBC_HGB_con_level | Routine blood examination: Level of average value of red blood cell (erythrocyte) HGB concentration | factor |
| ARBCV_value | Routine blood examination: Average value of red blood cell volume | numeric |
| ARBCV_level | Routine blood examination: Level of average value of red blood cell volume | factor |
| POB_value | Routine blood examination: Percentage value of basophil | numeric |
| POB_level | Routine blood examination: Level of percentage value of basophil | factor |
| POE_value | Routine blood examination: Percentage value of eosinophil | numeric |
| POE_level | Routine blood examination: Level of percentage value of eosinophil | factor |
| hemoglobin_value | Routine blood examination: Hemoglobin value | numeric |
| hemoglobin_level | Routine blood examination: Level of hemoglobin value | factor |
| PC_value | Routine blood examination: Platelet count | numeric |
| PC_level | Routine blood examination: Level of platelet count | factor |
| PON_value | Routine blood examination: Percentage value of neutrophilic segmented granulocyte | numeric |
| PON_level | Routine blood examination: Level of percentage value of neutrophilic segmented granulocyte | factor |
| AON_value | Routine blood examination: Absolute value of neutrophilic segmented granulocyte | numeric |
| AON_level | Routine blood examination: Level of absolute value of neutrophilic segmented granulocyte | factor |
| ALA_value | Routine biochemical examination: Alanine aminotransferase | numeric |
| ALA_level | Routine biochemical examination: Level of alanine aminotransferase | factor |
| ASA_value | Routine biochemical examination: Aspartate aminotransferase | numeric |
| ASA_level | Routine biochemical examination: Level of aspartate aminotransferase | factor |
| CK_value | Routine biochemical examination: Creatine kinase | numeric |
| CK_level | Routine biochemical examination: Level of creatine kinase | factor |
| LD_value | Routine biochemical examination: Lactate dehydrogenase | numeric |
| LD_level | Routine biochemical examination: Level of lactate dehydrogenase | factor |
| urea_value | Routine biochemical examination: Urea | numeric |
| urea_level | Routine biochemical examination: Level of urea | factor |
| DB_value | Routine biochemical examination: Direct bilirubin | numeric |
| DB_level | Routine biochemical examination: Level of direct bilirubin | factor |
| IDB_value | Routine biochemical examination: Indirect bilirubin | numeric |
| IDB_level | Routine biochemical examination: Level of indirect bilirubin | factor |
| TP_value | Routine biochemical examination: Total protein | numeric |
| TP_level | Routine biochemical examination: Level of total protein | factor |
| albumin_value | Routine biochemical examination: Albumin | numeric |
| albumin_level | Routine biochemical examination: Level of albumin | factor |
| creatinine_value | Routine biochemical examination: Creatinine | numeric |
| creatinine_level | Routine biochemical examination: Level of creatinine | factor |
| glucose_value | Routine biochemical examination: Glucose | numeric |
| glucose_level | Routine biochemical examination: Level of glucose | factor |
| AP_value | Routine biochemical examination: Alkaline phosphatase | numeric |
| AP_level | Routine biochemical examination: Level of alkaline phosphatase | factor |
| GT_value | Routine biochemical examination: Glutamyl transpeptidase | numeric |
| GT_level | Routine biochemical examination: Level of glutamyl transpeptidase | factor |
| sodium_value | Routine biochemical examination: Sodium | numeric |
| sodium_level | Routine biochemical examination: Level of sodium | factor |
| potassium_value | Routine biochemical examination: Potassium | numeric |
| potassium_level | Routine biochemical examination: Level of potassium | factor |
| chlorine_value | Routine biochemical examination: Chlorine | numeric |
| chlorine_level | Routine biochemical examination: Level of chlorine | factor |
| globulin_value | Routine biochemical examination: Globulin | numeric |
| globulin_level | Routine biochemical examination: Level of globulin | factor |
| WBR_value | Routine biochemical examination: White ball ratio | numeric |
| WBR_level | Routine biochemical examination: Level of white ball ratio | factor |
| UA_value | Routine biochemical examination: Uric acid | numeric |
| UA_level | Routine biochemical examination: Level of uric acid | factor |
| HD_value | Routine biochemical examination: Hydroxybutyrate dehydrogenase | numeric |
| HD_level | Routine biochemical examination: Level of hydroxybutyrate dehydrogenase | factor |
| cholesterol_value | Routine biochemical examination: Cholesterol | numeric |
| cholesterol_level | Routine biochemical examination: Level of cholesterol | factor |
| HDL_value | Routine biochemical examination: High density lipoprotein | numeric |
| HDL_level | Routine biochemical examination: Level of high density lipoprotein | factor |
| LDL_value | Routine biochemical examination: Low density lipoprotein | numeric |
| LDL_level | Routine biochemical examination: Level of low density lipoprotein | factor |
| cystatin C_value | Routine biochemical examination: Serum cystatin C | numeric |
| cystatin C_level | Routine biochemical examination: Level of serum cystatin C | factor |
| calcium_value | Routine biochemical examination: Calcium | numeric |
| calcium_level | Routine biochemical examination: Level of calcium | factor |
| magnesium_value | Routine biochemical examination:Magnesium | numeric |
| magnesium_level | Routine biochemical examination: Level of magnesium | factor |
| SIP_value | Routine biochemical examination: Serum inorganic phosphorus | numeric |
| SIP_level | Routine biochemical examination: Level of serum inorganic phosphorus | factor |
| CO2CP_value | Routine biochemical examination: Carbon dioxide combining power | numeric |
| CO2CP_level | Routine biochemical examination: Level of carbon dioxide combining power | factor |
| AG_value | Routine biochemical examination: Anion gap | numeric |
| AG_level | Routine biochemical examination: Level of aninon gap | factor |
| path.cast_value | Routine urine and stool examination: Pathocast | numeric |
| bacteria_value | Routine urine and stool examination: Bacteria | numeric |
| cast_value | Routine urine and stool examination: Cast | numeric |
| PH_value | Routine urine and stool examination: PH value | numeric |
| proportion_value | Routine urine and stool examination: Proportion value | numeric |
| conductivity_value | Routine urine and stool examination: Conductivity | numeric |
| EC_value | Routine urine and stool examination: Epithelial Cells | numeric |
| RC.ulsex_value | Routine urine and stool examination: RBC ulsex | numeric |
| mucus_value | Routine urine and stool examination: Mucus | factor |
| urobilinogen_value | Routine urine and stool examination: Urobilinogen | factor |
| NEC_value | Routine urine and stool examination: Normal epithelial Cells | factor |
| UOB_value | Routine urine and stool examination: Urine occult blood | factor |
| nitrite_value | Routine urine and stool examination: Nitrite | factor |
| ketone_value | Routine urine and stool examination: Ketone | factor |
| urine.bilirubin_value | Routine urine and stool examination: Urine bilirubin | factor |

- 1. **Additional analyses to assess the effect of confounders**

Table S2 Differences of 35 patients first diagnosed with BD depressive episodes and subsequently diagnosed with BD manic episodes.

|  | level | BD manic episodes | BD depressive episodes | p |
| --- | --- | --- | --- | --- |
| n |  | 35 | 35 |  |
| ALA_value (median [IQR]) | | 20.00 [14.00, 36.50] | 18.00 [12.50, 26.00] | 0.445 |
| ALA_level (%) | h | 4 (11.4) | 5 (14.3) | 1 |
|  | z | 31 (88.6) | 30 (85.7) |  |
| ASA_value (median [IQR]) | | 21.00 [18.00, 30.50] | 19.00 [15.00, 23.50] | 0.027 |
| ASA_level (%) | h | 3 (8.6) | 1 (2.9) | 0.607 |
|  | z | 32 (91.4) | 34 (97.1) |  |
| CK_value (median [IQR]) | | 91.00 [65.50, 242.50] | 77.00 [48.50, 116.50] | 0.049 |
| CK_level (%) | h | 11 (31.4) | 3 (8.6) | 0.036 |
|  | z | 24 (68.6) | 32 (91.4) |  |
| LD_value (median [IQR]) | | 165.00 [142.50, 237.00] | 148.00 [136.00, 172.50] | 0.048 |
| LD_level (%) | h | 10 (28.6) | 2 (5.7) | 0.039 |
|  | l | 1 (2.9) | 1 (2.9) |  |
|  | z | 24 (68.6) | 32 (91.4) |  |
| urea_value (median [IQR]) | | 4.80 [3.92, 6.60] | 4.31 [3.58, 5.45] | 0.114 |
| urea_level (%) | h | 5 (14.3) | 0 (0.0) | 0.066 |
|  | l | 3 (8.6) | 3 (8.6) |  |
|  | z | 27 (77.1) | 32 (91.4) |  |
| TB_value (median [IQR]) | | 8.40 [6.80, 12.25] | 9.50 [7.95, 11.70] | 0.681 |
| TB_level (%) | h | 1 (2.9) | 0 (0.0) | 0.602 |
|  | l | 2 (5.7) | 2 (5.7) |  |
|  | z | 32 (91.4) | 33 (94.3) |  |
| DB_value (median [IQR]) | | 3.20 [2.40, 4.20] | 3.10 [2.70, 4.10] | 0.972 |
| DB_level (%) | h | 2 (5.7) | 0 (0.0) | 0.473 |
|  | z | 33 (94.3) | 35 (100.0) |  |
| IDB_value (median [IQR]) | | 5.20 [4.25, 8.00] | 6.20 [5.00, 7.55] | 0.466 |
| IDB_level (%) | z | 35 (100.0) | 35 (100.0) | NA |
| TP_value (median [IQR]) | | 68.60 [65.05, 72.10] | 67.70 [63.60, 70.50] | 0.298 |
| TP_level (%) | l | 9 (25.7) | 10 (28.6) | 1 |
|  | z | 26 (74.3) | 25 (71.4) |  |
| albumin_value (median [IQR]) | | 43.60 [39.95, 46.60] | 43.50 [41.30, 44.90] | 0.626 |
| albumin_level (%) | l | 9 (25.7) | 3 (8.6) | 0.113 |
|  | z | 26 (74.3) | 32 (91.4) |  |
| creatinine_value (median [IQR]) | | 61.00 [55.00, 75.50] | 63.00 [54.00, 72.00] | 0.814 |
| creatinine_level (%) | z | 35 (100.0) | 35 (100.0) | NA |
| glucose_value (median [IQR]) | | 5.02 [4.63, 5.66] | 5.04 [4.46, 5.58] | 0.651 |
| glucose_level (%) | h | 6 (17.1) | 6 (17.1) | 0.355 |
|  | l | 2 (5.7) | 0 (0.0) |  |
|  | z | 27 (77.1) | 29 (82.9) |  |
| AP_value (median [IQR]) | | 71.00 [60.00, 88.50] | 63.00 [57.50, 80.00] | 0.219 |
| AP_level (%) | h | 1 (2.9) | 1 (2.9) | 1 |
|  | l | 1 (2.9) | 1 (2.9) |  |
|  | z | 33 (94.3) | 33 (94.3) |  |
| GT_value (median [IQR]) | | 19.00 [12.00, 23.50] | 15.00 [10.00, 20.50] | 0.151 |
| GT_level (%) | h | 3 (8.6) | 3 (8.6) | 1 |
|  | z | 32 (91.4) | 32 (91.4) |  |
| sodium_value (median [IQR]) | | 141.90 [140.25, 143.90] | 142.10 [140.30, 143.75] | 0.842 |
| sodium_level (%) | h | 0 (0.0) | 1 (2.9) | 0.368 |
|  | l | 1 (2.9) | 0 (0.0) |  |
|  | z | 34 (97.1) | 34 (97.1) |  |
| potassium_value (median [IQR]) | | 3.86 [3.75, 4.12] | 3.98 [3.79, 4.14] | 0.597 |
| potassium_level (%) | l | 4 (11.4) | 3 (8.6) | 1 |
|  | z | 31 (88.6) | 32 (91.4) |  |
| chlorine_value (median [IQR]) | | 104.50 [102.10, 106.95] | 105.30 [103.20, 106.95] | 0.511 |
| chlorine_level (%) | h | 0 (0.0) | 2 (5.7) | 0.356 |
|  | l | 3 (8.6) | 3 (8.6) |  |
|  | z | 32 (91.4) | 30 (85.7) |  |
| globulin_value (median [IQR]) | | 25.50 [23.25, 27.10] | 23.90 [21.75, 26.60] | 0.272 |
| globulin_level (%) | l | 1 (2.9) | 4 (11.4) | 0.353 |
|  | z | 34 (97.1) | 31 (88.6) |  |
| WBR_value (median [IQR]) | | 1.73 [1.58, 1.96] | 1.77 [1.58, 2.01] | 0.541 |
| WBR_level (%) | h | 1 (2.9) | 0 (0.0) | 1 |
|  | z | 34 (97.1) | 35 (100.0) |  |
| UA_value (median [IQR]) | | 327.00 [276.50, 384.50] | 291.00 [240.00, 323.50] | 0.012 |
| UA_level (%) | h | 7 (20.0) | 3 (8.6) | 0.375 |
|  | l | 2 (5.7) | 3 (8.6) |  |
|  | z | 26 (74.3) | 29 (82.9) |  |
| HD_value (median [IQR]) | | 133.00 [114.00, 174.50] | 117.00 [105.50, 141.00] | 0.04 |
| HD_level (%) | h | 8 (22.9) | 2 (5.7) | 0.088 |
|  | z | 27 (77.1) | 33 (94.3) |  |
| cholessterol_value (median [IQR]) | | 4.59 [3.90, 5.14] | 4.11 [3.69, 4.83] | 0.124 |
| cholessterol_level (%) | h | 1 (2.9) | 3 (8.6) | 0.607 |
|  | z | 34 (97.1) | 32 (91.4) |  |
| HDL_value (median [IQR]) | | 1.39 [1.14, 1.66] | 1.46 [1.14, 1.63] | 0.86 |
| HDL_level (%) | l | 4 (11.4) | 3 (8.6) | 1 |
|  | z | 31 (88.6) | 32 (91.4) |  |
| LDL_value (median [IQR]) | | 2.42 [2.02, 2.98] | 2.39 [1.94, 2.78] | 0.492 |
| LDL_level (%) | h | 0 (0.0) | 1 (2.9) | 1 |
|  | z | 35 (100.0) | 34 (97.1) |  |
| cystatinC_value (median [IQR]) | | 0.88 [0.78, 0.94] | 0.86 [0.74, 0.97] | 0.977 |
| cystatinC_level (%) | h | 4 (11.4) | 2 (5.7) | 0.431 |
|  | l | 0 (0.0) | 1 (2.9) |  |
|  | z | 31 (88.6) | 32 (91.4) |  |
| calcium_value (median [IQR]) | | 2.26 [2.18, 2.35] | 2.27 [2.17, 2.32] | 0.747 |
| calcium_level (%) | l | 2 (5.7) | 2 (5.7) | 1 |
|  | z | 33 (94.3) | 33 (94.3) |  |
| magnesium_value (median [IQR]) | | 0.90 [0.84, 0.96] | 0.87 [0.82, 0.92] | 0.101 |
| magnesium_level (%) | h | 1 (2.9) | 0 (0.0) | 1 |
|  | z | 34 (97.1) | 35 (100.0) |  |
| SIP_value (median [IQR]) | | 1.06 [0.92, 1.31] | 1.14 [0.98, 1.26] | 0.634 |
| SIP_level (%) | h | 2 (5.7) | 2 (5.7) | 1 |
|  | l | 2 (5.7) | 2 (5.7) |  |
|  | z | 31 (88.6) | 31 (88.6) |  |
| CO2CP_value (median [IQR]) | | 22.90 [21.35, 24.65] | 23.80 [21.55, 25.65] | 0.463 |
| CO2CP_level (%) | h | 1 (2.9) | 3 (8.6) | 0.323 |
|  | l | 1 (2.9) | 3 (8.6) |  |
|  | z | 33 (94.3) | 29 (82.9) |  |
| AG_value (median [IQR]) | | 18.80 [16.30, 20.10] | 18.00 [15.55, 19.75] | 0.353 |
| AG_level (%) | h | 9 (25.7) | 8 (22.9) | 0.822 |
|  | l | 1 (2.9) | 2 (5.7) |  |
|  | z | 25 (71.4) | 25 (71.4) |  |
| RBC_CV_value (median [IQR]) | | 13.20 [12.80, 13.85] | 13.70 [12.95, 14.20] | 0.427 |
| RBC_CV_level (%) | high | 4 (11.4) | 5 (14.3) | 1 |
|  | normal | 31 (88.6) | 30 (85.7) |  |
| RBC_SD_value (median [IQR]) | | 45.20 [43.60, 47.70] | 46.20 [43.50, 48.95] | 0.401 |
| RBC_SD_level (%) | high | 2 (5.7) | 1 (2.9) | 0.84 |
|  | low | 1 (2.9) | 1 (2.9) |  |
|  | normal | 32 (91.4) | 33 (94.3) |  |
| WCC_value (median [IQR]) | | 7.50 [5.85, 9.15] | 5.90 [5.20, 6.95] | 0.014 |
| WCC_level (%) | high | 9 (25.7) | 3 (8.6) | 0.113 |
|  | normal | 26 (74.3) | 32 (91.4) |  |
| POM_value (median [IQR]) | | 6.70 [5.65, 7.95] | 5.80 [5.35, 6.95] | 0.063 |
| POM_level (%) | high | 3 (8.6) | 1 (2.9) | 0.607 |
|  | normal | 32 (91.4) | 34 (97.1) |  |
| AOM_value (median [IQR]) | | 0.50 [0.40, 0.70] | 0.40 [0.30, 0.50] | 0.003 |
| AOM_level (%) | high | 12 (34.3) | 2 (5.7) | 0.007 |
|  | normal | 23 (65.7) | 33 (94.3) |  |
| RBCC_value (median [IQR]) | | 4.40 [4.10, 4.70] | 4.30 [4.05, 4.75] | 0.841 |
| RBCC_level (%) | high | 1 (2.9) | 1 (2.9) | 0.923 |
|  | low | 4 (11.4) | 3 (8.6) |  |
|  | normal | 30 (85.7) | 31 (88.6) |  |
| hematocrit_value (median [IQR]) | | 0.40 [0.40, 0.40] | 0.40 [0.40, 0.40] | 0.416 |
| hematocrit_level (%) | low | 3 (8.6) | 3 (8.6) | 1 |
|  | normal | 32 (91.4) | 32 (91.4) |  |
| POL_value (median [IQR]) | | 26.30 [15.85, 39.25] | 29.70 [20.55, 37.30] | 0.382 |
| POL_level (%) | high | 1 (2.9) | 2 (5.7) | 0.641 |
|  | low | 11 (31.4) | 8 (22.9) |  |
|  | normal | 23 (65.7) | 25 (71.4) |  |
| AOL_value (median [IQR]) | | 2.00 [1.25, 2.65] | 1.70 [1.45, 2.25] | 0.733 |
| AOL_level (%) | high | 4 (11.4) | 3 (8.6) | 0.668 |
|  | low | 5 (14.3) | 3 (8.6) |  |
|  | normal | 26 (74.3) | 29 (82.9) |  |
| ARBC_HGB_value (median [IQR]) | | 30.50 [29.50, 32.05] | 31.00 [29.55, 31.55] | 0.953 |
| ARBC_HGB_level (%) | high | 2 (5.7) | 2 (5.7) | 1 |
|  | low | 1 (2.9) | 1 (2.9) |  |
|  | normal | 32 (91.4) | 32 (91.4) |  |
| ARBC_HGB_con_value (median [IQR]) | | 329.00 [323.00, 341.00] | 327.00 [323.50, 333.50] | 0.452 |
| ARBC_HGB_con_level (%) | high | 0 (0.0) | 1 (2.9) | 0.602 |
|  | low | 3 (8.6) | 3 (8.6) |  |
|  | normal | 32 (91.4) | 31 (88.6) |  |
| ARBCV_value (median [IQR]) | | 92.40 [89.05, 97.10] | 93.50 [90.30, 95.90] | 0.851 |
| ARBCV_level (%) | high | 4 (11.4) | 1 (2.9) | 0.379 |
|  | low | 1 (2.9) | 1 (2.9) |  |
|  | normal | 30 (85.7) | 33 (94.3) |  |
| POB_value (median [IQR]) | | 0.40 [0.20, 0.50] | 0.40 [0.20, 0.50] | 0.642 |
| POB_level (%) | high | 0 (0.0) | 1 (2.9) | 1 |
|  | normal | 35 (100.0) | 34 (97.1) |  |
| POE_value (median [IQR]) | | 1.50 [0.90, 2.40] | 1.70 [1.00, 2.50] | 0.828 |
| POE_level (%) | low | 3 (8.6) | 2 (5.7) | 1 |
|  | normal | 32 (91.4) | 33 (94.3) |  |
| hemoglobin_value (median [IQR]) | | 134.00 [124.00, 142.00] | 130.00 [125.00, 146.00] | 0.897 |
| hemoglobin_level (%) | low | 3 (8.6) | 5 (14.3) | 0.707 |
|  | normal | 32 (91.4) | 30 (85.7) |  |
| PC_value (median [IQR]) | | 235.00 [176.00, 264.00] | 188.00 [150.00, 236.00] | 0.148 |
| PC_level (%) | high | 5 (14.3) | 4 (11.4) | 0.801 |
|  | low | 1 (2.9) | 2 (5.7) |  |
|  | normal | 29 (82.9) | 29 (82.9) |  |
| PON_value (median [IQR]) | | 63.10 [51.60, 74.80] | 62.20 [52.65, 70.15] | 0.481 |
| PON_level (%) | high | 8 (22.9) | 5 (14.3) | 0.65 |
|  | low | 2 (5.7) | 2 (5.7) |  |
|  | normal | 25 (71.4) | 28 (80.0) |  |
| AON_value (median [IQR]) | | 4.60 [3.15, 6.70] | 3.50 [2.75, 4.15] | 0.067 |
| AON_level (%) | high | 11 (31.4) | 5 (14.3) | 0.156 |
|  | low | 0 (0.0) | 1 (2.9) |  |
|  | normal | 24 (68.6) | 29 (82.9) |  |
| gender (%) | F | 21 (60.0) | 21 (60.0) | 1 |
|  | M | 14 (40.0) | 14 (40.0) |  |
| age (median [IQR]) | | 44.00 [29.50, 52.50] | 42.00 [27.50, 50.50] | 0.522 |
| age.group (%) | 0-17 | 1 (2.9) | 1 (2.9) | 0.989 |
|  | 18-35 | 13 (37.1) | 13 (37.1) |  |
|  | 36-60 | 15 (42.9) | 16 (45.7) |  |
|  | 61- | 6 (17.1) | 5 (14.3) |  |
| job.status (%) | basic | 4 (11.4) | 6 (17.1) | 0.974 |
|  | freelance | 3 (8.6) | 3 (8.6) |  |
|  | labor | 3 (8.6) | 3 (8.6) |  |
|  | mangement | 3 (8.6) | 3 (8.6) |  |
|  | other | 6 (17.1) | 7 (20.0) |  |
|  | retired | 10 (28.6) | 7 (20.0) |  |
|  | service | 0 (0.0) | 1 (2.9) |  |
|  | student | 3 (8.6) | 3 (8.6) |  |
|  | unemployed | 3 (8.6) | 2 (5.7) |  |
| marital.status (%) | divorced | 3 (8.6) | 4 (11.4) | 0.961 |
|  | married | 20 (57.1) | 18 (51.4) |  |
|  | single | 10 (28.6) | 11 (31.4) |  |
|  | widow | 2 (5.7) | 2 (5.7) |  |
| ethic (%) | Han | 35 (100.0) | 35 (100.0) | NA |
| type (%) | self-paid | 35 (100.0) | 35 (100.0) | NA |
| pay.type (%) | cash | 13 (37.1) | 14 (40.0) | 0.694 |
|  | city coverage | 18 (51.4) | 19 (54.3) |  |
|  | province coverage | 4 (11.4) | 2 (5.7) |  |
| source (%) | city | 23 (65.7) | 23 (65.7) | 0.881 |
|  | in province | 10 (28.6) | 9 (25.7) |  |
|  | out province | 2 (5.7) | 3 (8.6) |  |
| if.trans (%) | N | 32 (91.4) | 32 (91.4) | 1 |
|  | Y | 3 (8.6) | 3 (8.6) |  |
| main.diag.in.match (%) | 0 | 0 (0.0) | 1 (2.9) | 1 |
|  | 1 | 35 (100.0) | 34 (97.1) |  |
| comorbidity.number (median [IQR]) | | 0.00 [0.00, 2.00] | 0.00 [0.00, 2.00] | 0.854 |
| psychiatric.comorbidity (%) | N | 33 (94.3) | 34 (97.1) | 1 |
|  | Y | 2 (5.7) | 1 (2.9) |  |
| psychiatric.comorbidity.number (median [IQR]) | | 0.00 [0.00, 0.00] | 0.00 [0.00, 0.00] | 0.558 |
| endocrine.comorbidity (%) | N | 26 (74.3) | 28 (80.0) | 0.776 |
|  | Y | 9 (25.7) | 7 (20.0) |  |
| endocrine.comorbidity.number (median [IQR]) | | 0.00 [0.00, 0.50] | 0.00 [0.00, 0.00] | 0.547 |
| nerve.comorbidity (%) | N | 34 (97.1) | 34 (97.1) | 1 |
|  | Y | 1 (2.9) | 1 (2.9) |  |
| nerve.comorbidity.number (median [IQR]) | | 0.00 [0.00, 0.00] | 0.00 [0.00, 0.00] | 0.984 |
| digestive.comorbidity (%) | N | 30 (85.7) | 33 (94.3) | 0.426 |
|  | Y | 5 (14.3) | 2 (5.7) |  |
| digestive.comorbidity.number (median [IQR]) | | 0.00 [0.00, 0.00] | 0.00 [0.00, 0.00] | 0.21 |
| circulatory.comorbidity (%) | N | 30 (85.7) | 29 (82.9) | 1 |
|  | Y | 5 (14.3) | 6 (17.1) |  |
| circulatory.comorbidity.number (median [IQR]) | | 0.00 [0.00, 0.00] | 0.00 [0.00, 0.00] | 0.744 |
| respiratory.comorbidity (%) | N | 32 (91.4) | 33 (94.3) | 1 |
|  | Y | 3 (8.6) | 2 (5.7) |  |
| respiratory.comorbidity.number (median [IQR]) | | 0.00 [0.00, 0.00] | 0.00 [0.00, 0.00] | 0.645 |
| cancer.comorbidity (%) | N | 33 (94.3) | 34 (97.1) | 1 |
|  | Y | 2 (5.7) | 1 (2.9) |  |
| cancer.comorbidity.number (median [IQR]) | | 0.00 [0.00, 0.00] | 0.00 [0.00, 0.00] | 0.558 |
| temperature (median [IQR]) | | 36.50 [36.40, 36.60] | 36.50 [36.40, 36.60] | 0.556 |
| pulse (median [IQR]) | | 89.00 [79.00, 101.00] | 85.00 [74.50, 92.00] | 0.137 |
| breathing (median [IQR]) | | 20.00 [20.00, 20.00] | 20.00 [20.00, 20.00] | 0.759 |
| systolic.pressure (median [IQR]) | | 120.58 [116.00, 127.50] | 115.00 [108.00, 130.00] | 0.198 |
| diastolic.pressure (median [IQR]) | | 77.45 [71.00, 83.50] | 76.00 [69.00, 83.50] | 0.613 |
| surg.no (median [IQR]) | | 0.00 [0.00, 0.00] | 0.00 [0.00, 0.00] | 0.293 |
| PH.relianility (%) | almost reliable | 1 (2.9) | 6 (17.1) | 0.111 |
|  | reliable | 34 (97.1) | 29 (82.9) |  |
| hometown (%) | Chongqing | 2 (5.7) | 2 (5.7) | 0.938 |
|  | other | 5 (14.3) | 4 (11.4) |  |
|  | Sichuang | 28 (80.0) | 29 (82.9) |  |
| surgery.his (%) | N | 26 (74.3) | 25 (71.4) | 1 |
|  | Y | 9 (25.7) | 10 (28.6) |  |
| allergy (%) | N | 26 (74.3) | 27 (77.1) | 1 |
|  | Y | 9 (25.7) | 8 (22.9) |  |
| blood.trans (%) | N | 35 (100.0) | 33 (94.3) | 0.473 |
|  | Y | 0 (0.0) | 2 (5.7) |  |
| durg.his (%) | none | 20 (57.1) | 23 (65.7) | 0.24 |
|  | often | 11 (31.4) | 9 (25.7) |  |
|  | sometimes | 4 (11.4) | 1 (2.9) |  |
|  | unknown | 0 (0.0) | 2 (5.7) |  |
| nutrition (%) | good | 32 (91.4) | 35 (100.0) | 0.238 |
|  | medium | 3 (8.6) | 0 (0.0) |  |
| cooperation (%) | FALSE | 4 (11.4) | 0 (0.0) | 0.122 |
|  | TRUE | 31 (88.6) | 35 (100.0) |  |
| mooddown (%) | FALSE | 3 (8.6) | 4 (11.4) | 1 |
|  | TRUE | 32 (91.4) | 31 (88.6) |  |
| moodup (%) | FALSE | 7 (20.0) | 11 (31.4) | 0.412 |
|  | TRUE | 28 (80.0) | 24 (68.6) |  |
| moodunstable (%) | FALSE | 0 (0.0) | 4 (11.4) | 0.122 |
|  | TRUE | 35 (100.0) | 31 (88.6) |  |
| sleepbad (%) | FALSE | 31 (88.6) | 30 (85.7) | 1 |
|  | TRUE | 4 (11.4) | 5 (14.3) |  |
| provoke (%) | FALSE | 28 (80.0) | 32 (91.4) | 0.306 |
|  | TRUE | 7 (20.0) | 3 (8.6) |  |
| worry (%) | FALSE | 32 (91.4) | 28 (80.0) | 0.306 |
|  | TRUE | 3 (8.6) | 7 (20.0) |  |
| talking (%) | FALSE | 19 (54.3) | 28 (80.0) | 0.042 |
|  | TRUE | 16 (45.7) | 7 (20.0) |  |
| suicide (%) | FALSE | 34 (97.1) | 34 (97.1) | 1 |
|  | TRUE | 1 (2.9) | 1 (2.9) |  |
| pain (%) | FALSE | 35 (100.0) | 35 (100.0) | NA |
| eating (%) | FALSE | 35 (100.0) | 35 (100.0) | NA |
| hearing (%) | FALSE | 35 (100.0) | 35 (100.0) | NA |
| relapse (%) | FALSE | 17 (48.6) | 16 (45.7) | 1 |
|  | TRUE | 18 (51.4) | 19 (54.3) |  |
| worsen (%) | FALSE | 30 (85.7) | 29 (82.9) | 1 |
|  | TRUE | 5 (14.3) | 6 (17.1) |  |


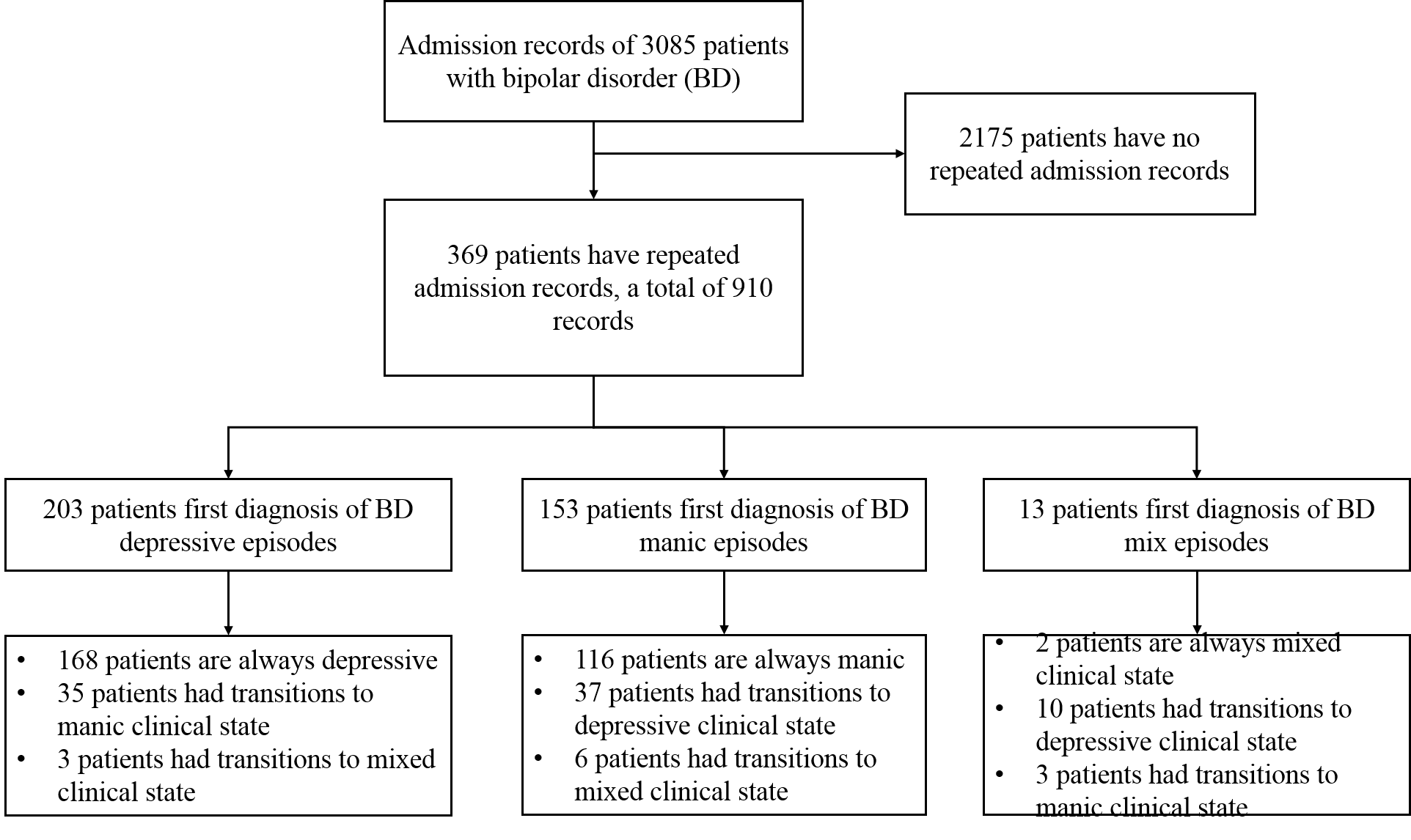


Figure S4 (a) The flow of longitudinal cohort construction.


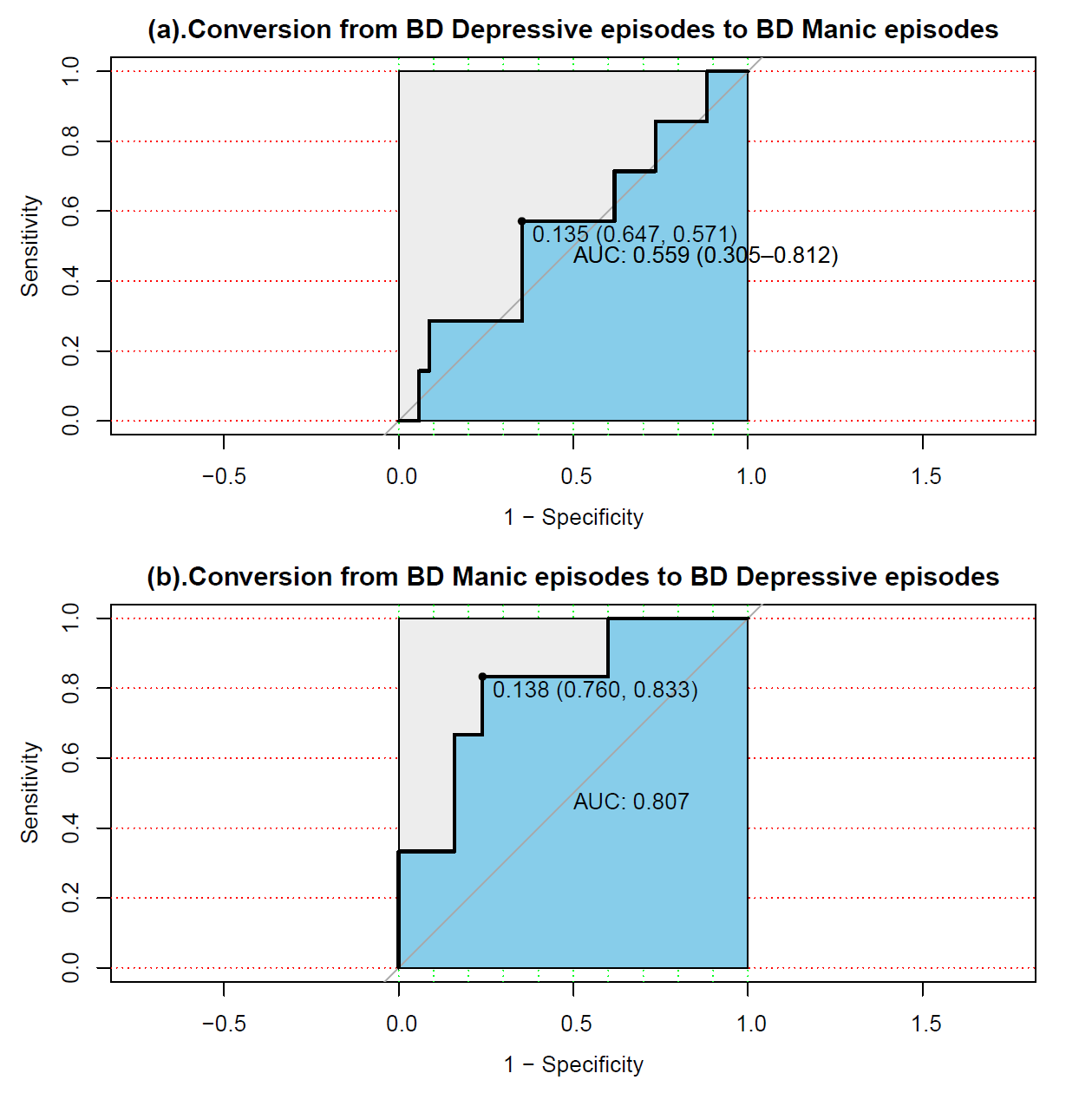


Figure S4 (b) ROC curves for predicting the longitudinal evolution of different BD clinical states.
